# Flexible and Long-Lived Naphthalene Diimide-Based Photothermal Films with Rapid and Highly Cyclable Heat Conversion

**DOI:** 10.1021/acsapm.6c01376

**Published:** 2026-08-07

**Authors:** Ziyao Xu, Bart Dietrich, Katarzyna Kozioł, Emily R. Draper

**Affiliations:** † School of Chemistry, 3526 University of Glasgow, Glasgow G12 8QQ, U.K.; ‡ James Watt School of Engineering, 3526 University of Glasgow, Glasgow G12 8QQ, U.K.

**Keywords:** Naphthalene diimides, photothermal films, organic materials, pH, reduced species, long lifetime

## Abstract

Flexible photothermal materials have attracted considerable attention for applications in biomedicine, energy conversion, and smart devices. While state-of-the-art inorganic nanomaterials and conductive polymers exhibit excellent photothermal properties, simultaneously achieving efficient photothermal performance, environmental stability, and mechanical flexibility remains challenging. Naphthalene diimide (NDI)-based materials are well-known for their strong photothermal response resulting from the formation of radical anions. In this work, we employ glycerol as a plasticizer and humectant to improve film uniformity in NDI-based films, while the incorporation of PVA enhances long-term stability by slowing NDI oxidation and enabling mechanical tunability. Photothermal performance was evaluated using direct thermal imaging and Peltier voltage measurements. Under 617 nm irradiation at 16 mW cm^–2^, the NDI-I films achieved a maximum temperature increase of 7.6 °C (pH 5) and a peak Peltier output voltage of 0.0052 V (pH 6). The addition of glycerol and PVA significantly improved film homogeneity and mechanical durability. The resulting PVA/NDI films show remarkable oxidative stability, retaining their reduced state for over 7 days compared to only about 3 h for pristine NDI films. Furthermore, the films demonstrate excellent photothermal cycling stability, with no observable performance degradation after more than 500 irradiation cycles. These results highlight the potential of tailored PVA/NDI composite films for long-lasting, flexible photothermal applications.

## Graphical abstract

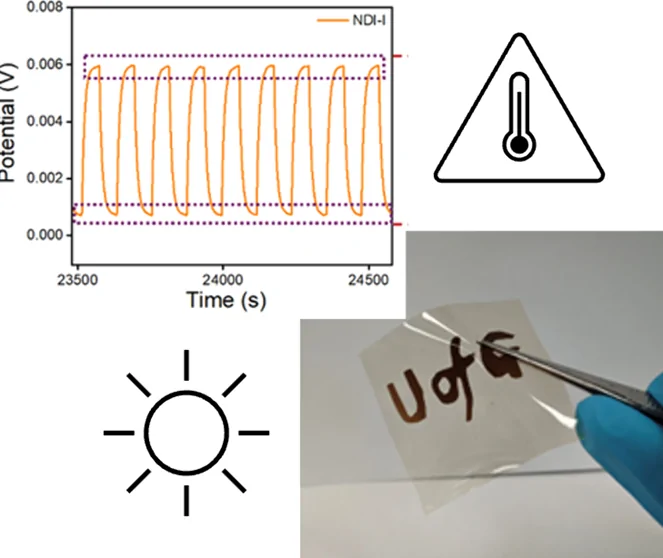

## Introduction

The photothermal effect is widely exploited in biomedical therapy, solar energy conversion, imaging, and environmental remediation.^1,2^ In these applications, photothermal films are particularly significant because they combine efficient light absorption, controllable thermal response, and good mechanical stability, making them suitable for scalable devices and flexible applications.

State-of-the-art photothermal materials include nanomaterials such as MXenes, graphene derivatives, transition-metal chalcogenides, and conductive polymers, as well as organic conjugated systems. These materials exhibit efficient light-to-heat conversion and have enabled significant advances in flexible photothermal devices.^3–5^ However, challenges associated with processability, environmental stability, mechanical durability, or manufacturing cost continue to motivate the development of alternative organic photothermal platforms.^6,7^ Organic photothermal materials offer tunable molecular structures in the form of different chromophores, ease of synthesis, and better processability, which allow integration into films and composites.^8–11^ Their photothermal behavior originates from the excitation of electrons upon light absorption and subsequent nonradiative relaxation, where the absorbed photon energy is dissipated as heat. This nonradiative pathway dominates in organic systems and governs their conversion efficiency.

Among organic chromophores, naphthalene diimides (NDIs) represent an important class of photothermal molecules because of their rigid aromatic core, electron-withdrawing imide groups, and strong electron-accepting characteristics. Their extended π-conjugation facilitates efficient charge delocalization, and the imide groups enable reversible reduction to stable radical anions and dianions.^10,11^ This redox activity is central to their photothermal behavior, since the formation and relaxation of their excited states dissipate energy as heat. Structural modification of NDIs can also enhance their visible and near-infrared (NIR) absorption. For example, introducing dendritic or anionic donor groups increases light absorption and promotes nonradiative decay pathways, resulting in higher photothermal conversion efficiencies.^12,13^ Supramolecular assembly provides additional tunability and can generate pH-, temperature-, or solvent-responsive heating behaviors. Recent studies have also shown that optimized NDI derivatives possess high thermal stability and good biocompatibility, making them promising candidates for photothermal therapy, environmental remediation, and other photothermal or photochemical applications.^14–18^ However, purely organic films can suffer from poor mechanical properties or nonuniformity.^19,20^ State-of-the-art organic photothermal systems, including conjugated polymers, rylene diimide derivatives, and conductive polymer films, have demonstrated efficient photothermal performance and good processability.^4,21,22^ Nevertheless, many studies focus primarily on maximizing photothermal output, whereas oxidative stability, long-term environmental durability, and mechanical robustness under repeated deformation remain less explored.

Glycerol is widely used as both an absorbent and a plasticizer to improve film flexibility, reduce brittleness, and promote smoother film formation.^23^ Zhou et al. showed that soaking hydrogels in glycerol through a one-pot solvent-displacement method significantly enhanced their flexibility and toughness, while also producing smoother surfaces due to the lubricating effect of glycerol.^24,25^ Similar improvements have been reported across various polymer systems: in starch, alginate, gelatin, and cellulose films, glycerol disrupts intermolecular hydrogen bonding and lowers the glass-transition temperature, leading to softer and more ductile films.^26,27^ In conductive films such as PEDOT:PSS, glycerol also enhances mechanical flexibility and prevents cracking during bending. Overall, glycerol is one of the most versatile and effective additives for producing flexible, uniform, and less brittle films.

To fabricate stable and flexible photothermal films, polymers are often incorporated as supporting matrices.^28,29^ Many active small molecules show poor film-forming ability, low mechanical strength, or limited solubility when used alone; embedding them in a polymer network addresses these limitations. Polymers act as film-forming agents and mechanical supports, improving adhesion to substrates and resistance to environmental stress. Commonly used polymers include polystyrene (PS), poly­(methyl methacrylate) (PMMA), polyethylene (PE), polyethylene terephthalate (PET), polyimides (PI), poly­(ethylene oxide) (PEO), and poly­(vinyl alcohol) (PVA).^30,31^ PVA and PEO are particularly useful for NDI-based systems because of their biocompatibility, solubility, and ability to form smooth, flexible films. Polymer incorporation enhances light and heat stability, prevents delamination or cracking, and maintains the photothermal response during repeated irradiation cycles. Optimizing polymer composition and active-material loading ensures homogeneous optical properties and avoids phase separation.

Overall, organic photothermal films, especially those integrating NDI derivatives with suitable polymer matrices, offer a promising platform for efficient and sustainable light-to-heat conversion. Their molecular tunability, mechanical flexibility, and processability make them strong candidates for applications in biomedical therapy, solar energy harvesting, and multifunctional photothermal devices. In this study, we focus on incorporating our NDI derivatives into flexible polymer matrices to develop reusable and mechanically robust photothermal films that combine efficient light-to-heat conversion with environmental stability and flexible operation. We show how film composition, molecular structure, and processing conditions influence photothermal efficiency, mechanical stability, and long-term cycling performance, evaluated by quantifying the films’ steady-state and cycling photothermal response, and determining the durability of the photothermal effect over multiple use cycles. Based on our previous solution-phase work, NDI-I (NDI appended with l-isoleucine) was identified as the most promising photothermal candidate, and therefore serves as a key material in our film-based investigations (Figure 1a).^32^ We then compare with a lesser performing NDI (NDI-F, Figure 1b) to examine whether the same holds true when processed into a film. This enables us to understand how much the active molecule influences the photothermal performance over the additives.

**Figure 1:**
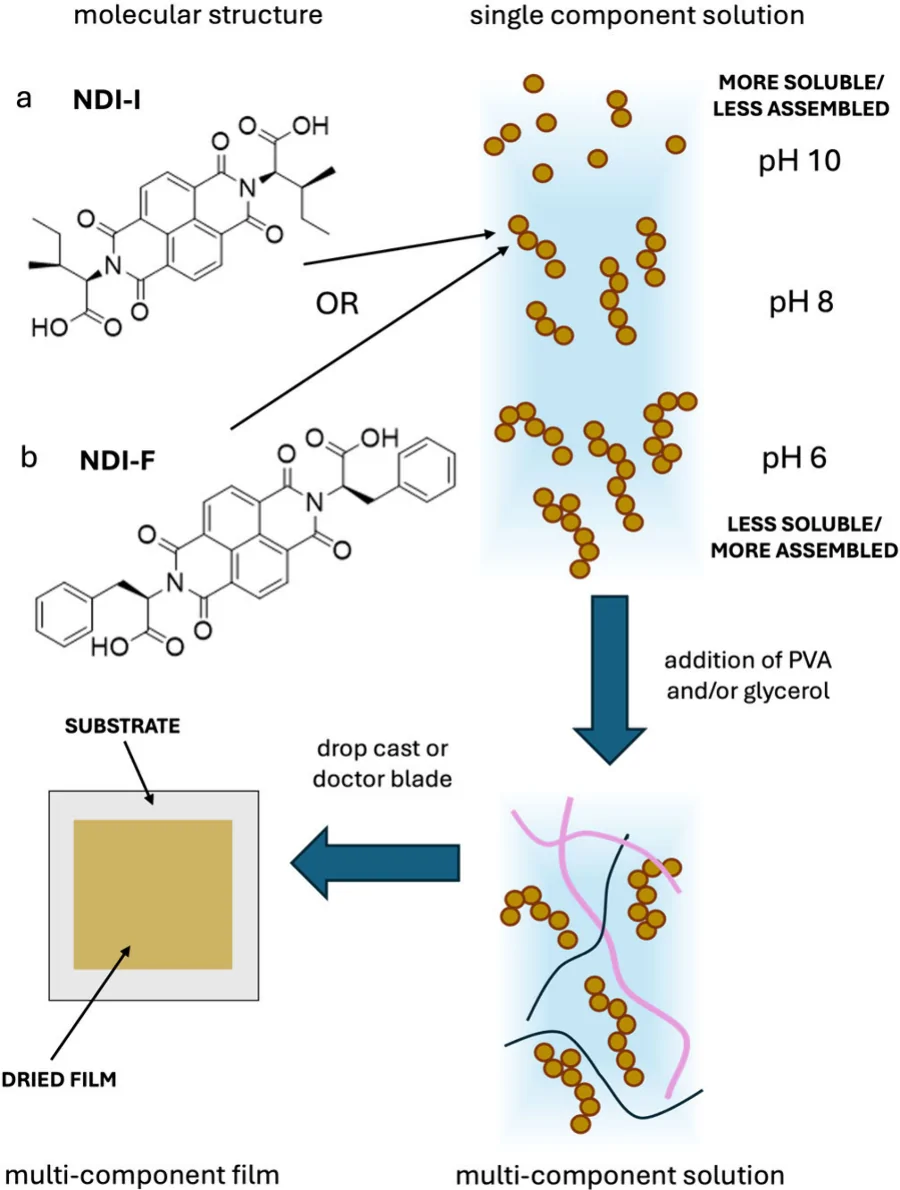
Cartoon showing the assembly and preparation of the films used in this study using (a) NDI-I and (b) NDI-F.

## Experimental Section

Naphthalene-1,4,5,8-tetracarboxylic acid dianhydride (NTCDA), imidazole, and l-isoleucine were purchased from Fluorochem. l-phenylalanine was purchased from Merck Life Science, and hydrochloric acid was purchased from Honeywell Fluka. PVA­(polyvinyl achohol, 87–89% hydrolyzed, Mw 31,000–50,000), PVP (polyvinylpyrrolidone, average Mw 40,000), and PEG (polyethylene glycol, average Mn 950–1,050) were purchased from Merck Life Science. Deionized water was used throughout.

### Measurements and Characterization

Photothermal properties were evaluated by monitoring heat generation using an infrared thermal camera and by measuring the corresponding electrical potential via a thermoelectric setup. Detailed procedures are provided in the Supporting Information. The surface morphology of the films was characterized using optical microscopy. The formation of reduced species was monitored by UV–Vis absoprtion spectroscopy. Mechanical properties of the films were measured using a nanoindenter. The viscosity of the solutions was determined using a rheometer.

## Results and Discussion

To investigate the film properties, films were first prepared from NDI-I solutions (10 mg mL^–1^) at three different pHs (6, 8, and 10) employing drop-casting. These pHs were chosen due to the different NDI-I assemblies found at those values (Figure 1).^32^ We wanted to investigate whether the differences seen in solution would persist upon drying and therefore affect photothermal behavior in the films.

First, optical microscopic analysis was used to assess the surface characteristics of the thin films. It is important that drying the films does not compromise their integrity (i.e., no cracks, crystallization, phase separation etc. upon drying). Images were collected at different magnifications from the middle and edge regions of each of the films prepared at pH 6, 8, and 10, and are shown in Figure S2, Figure S3, and Figure S4. Films from solutions at pH 6 and 10 exhibited numerous crystal-like structures on their surfaces. Beneath these surface features, clear cracks within the thin films were observed (as shown in Figure 2a,c). Conversely, thin films produced from the pH 8 solution (Figure 2b) only displayed surface cracks, with no observable crystallization. Crystal-like structures were observed on the surfaces of films prepared at pH 6 and pH 10 and may be associated with salt crystallization during the pH adjustment process. Therefore, NDI-I films at pH 8 were considered optimal for further testing as they showed the greatest uniformity.

**Figure 2:**
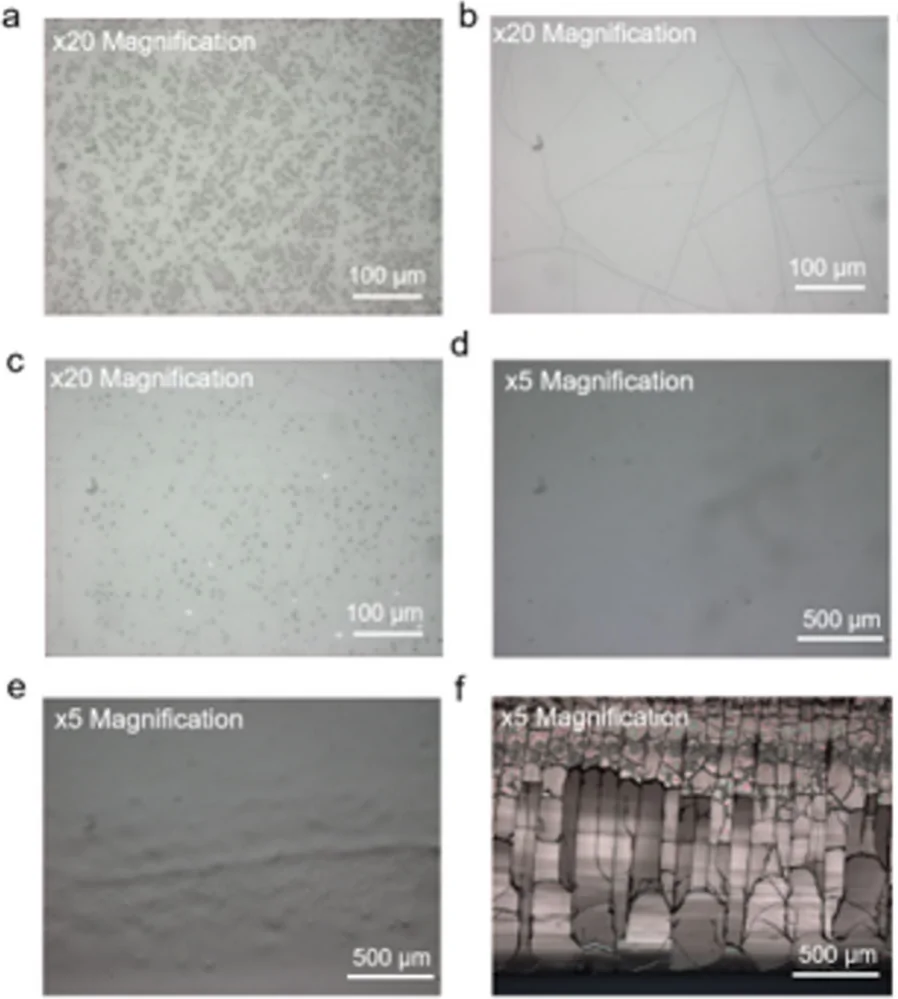
Optical microscopy images of NDI-I films on glass prepared at different pH values without glycerol: (a) pH 6, (b) pH 8, and (c) pH 10. (d) Image of the center of a film prepared at pH 8 with 0.4% glycerol. Images of film edges prepared (e) with glycerol (0.4%) and (f) without glycerol.

Next, we investigated doctor blade (Figure S5a) and drop-casting (Figure S5b) film preparation methods using pH 8 NDI-I solutions and varying annealing temperatures. As depicted in Figure S5, cracks appeared in the films prepared by both doctor blade and drop casting at annealing temperatures of 60, 70, and 80 °C. Interestingly, there were minimal differences between the two methods regardless of the annealing temperature used, suggesting that it is likely material composition rather than the process which is causing issues upon drying. The presence of cracks, likely resulting from insufficient water content in the films, can impede their uniformity. To address this issue, we added glycerol to act as a humectant. As shown in Figure 2 **d and 2e**, the inclusion of just 0.4% glycerol resulted in no visible cracks in the films.

While films prepared from pure NDI-I solutions exhibited a rough, crystalline surface morphology (Figure 2f), those incorporating 0.4% glycerol adhered well to the substrate and displayed a more homogeneous and uniform appearance (Figure 2e and **S6**). This improvement in film quality was also observed at other pH values and may be attributed to the humectant and nonvolatile plasticizing properties of glycerol, which retard water loss and mitigate crack formation during the drying process.^33,34^ The effect extends beyond the central region of the thin films to their edges, resulting in greater uniformity throughout.

With uniform films in hand, we were able to assess their photothermal behavior. To do this, the NDI films were irradiated with 365 nm light for 5 min to generate the reduced species, which remains stable for several hours. After the films had completely cooled, we irradiated them with 617 nm light. The reduced species has an absorption band in the visible region which permits it to absorb visible (in this case 617 nm) light and be evaluated for photothermal performance. By comparing the temperature rise from ambient of the irradiated NDI films and a control sample, we can calculate the temperature increase.^32^

Measurements were then carried out to compare the temperatures of irradiated thin films both with and without glycerol. Very little difference was observed between the two, indicating that the inclusion of glycerol does not compromise the photothermal properties of the thin films (see Figure S7).

To collect the thermal data, we typically use a thermal camera.^35,36^ However, several factors can lead to inaccurate results with this method. These include fluctuations in ambient temperature, the limited resolution of the thermal camera, and the time required to transfer samples out of the LED light box for imaging, during which heat may dissipate. To overcome these limitations, we developed a method that uses a Peltier module to measure the temperature increase of films. When an electric current is applied to a Peltier module, it acts as a heat pump moving heat from one side to the other. This is the Peltier effect and the usual use case of Peltier modules. When a temperature difference is applied to the module, however, it acts as a thermoelectric generator and a voltage appears on its terminals which is approximately proportional to the temperature difference. This is the Seebeck effect and the basis for our use case. With this method, we can continuously monitor the photothermal response during and after irradiation without the need to move the sample. Additionally, it offers high accuracy, as the generated voltage can be measured with high accuracy (see Table S1).

For testing, the NDI films were prepared on aluminum foil (better heat transfer to the Peltier module than glass), then attached to one side of the Peltier module (Figure 3a). A heat sink was attached to the other side using thermal paste to act as a thermal buffer: as the film side heats up, some of this heat may transfer through the internal structure of the Peltier to the cold side, thus decreasing the temperature difference between both sides and depressing the generated voltage. The heat sink adds thermal bulk to absorb “leaked” heat and is expected to keep the cold side closer to ambient temperature. A potentiostat was used to measure the generated electrical potential during irradiation (see Figure 3b). By comparing the voltage generated by the Peltier module with different films attached, we can evaluate the heat generated by each film and thereby assess their relative photothermal performance. Figure S8 shows an example of how the value was determined. When the 617 nm light was switched on, the potential began increasing and reached a plateau after approximately 1 min. The plateau value was recorded as the data point value. As shown in Figure S9, measurements without a heat sink exhibited high variability and no clear trend, indicating significant error. In contrast, using a heat sink reduced variability and produced more consistent voltage outputs from the Peltier module (Figure S9 and Figure 3d).

**Figure 3:**
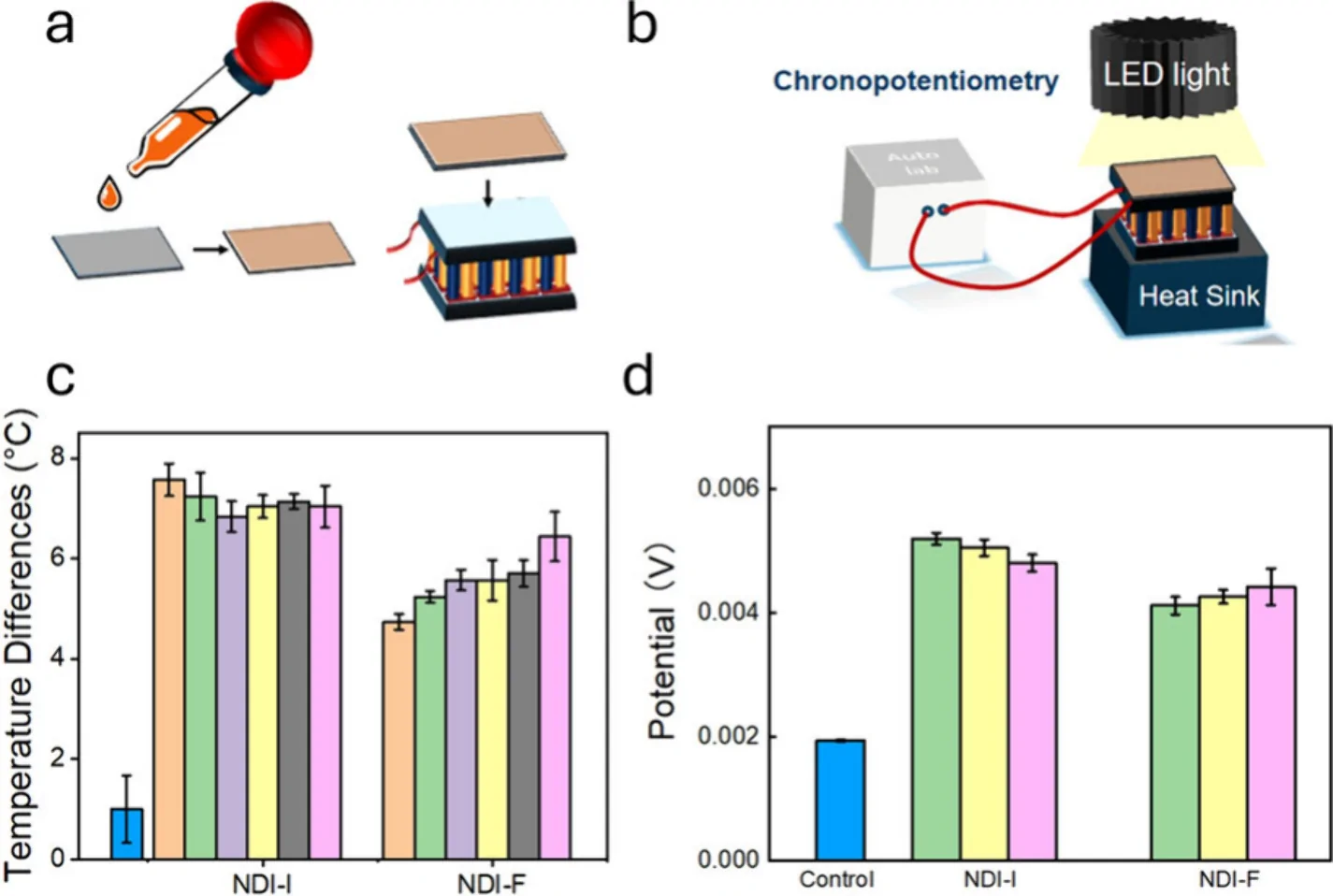
(a) Cartoon showing the preparation of NDI films on the Peltier module and (b) the measurement of a potential using a potentiostat. (c) Temperature increases above ambient of NDI-I and NDI-F films under irradiation. (d) Voltage output of the Peltier module with one side attached to NDI-I or NDI-F films (blue: control, orange: pH 5, green: pH 6, purple: pH 7, yellow: pH 8, gray: pH 9, and pink: pH 10). Error bars calculated from standard deviation of three measurements.

Next, we prepared NDI films and evaluated their photothermal performance using both the thermal imaging and Peltier module methods. Temperature differences were calculated by subtracting the ambient temperature from the sample temperature, allowing us to compare which films generated more heat. For voltage measurements, we applied the Peltier-based method described above and tested films prepared at pH 6, 8, and 10.

The films prepared with the addition of glycerol on aluminum foil (Figure S10) were smooth, flat, and highly homogeneous, which enabled more reliable and accurate measurements, unlike when prepared on a glass substrate. Films of NDI-I and NDI-F were prepared. According to our previous work, NDI-I solutions exhibit the highest photothermal performance among the investigated derivatives, whereas NDI-F shows comparatively lower performance.^32^

As pH can influence the assembly behavior of the solutions, we have investigated how pH affects their photothermal properties in a previous study.^32^ We therefore next examined the impact of pH on the photothermal properties of the corresponding films with the inclusion of glycerol. As the inclusion of glycerol improved the film quality at both lower and higher pHs, we could now study films prepared over a wider pH range (from pH 5 to pH 10). The thermal camera data (Figure 3c, Figure S11) under an irradiation intensity of 16 mW cm^–2^ show that the temperature differences among the NDI-I films were generally similar, with the film prepared at pH 5 exhibiting a slightly higher temperature rise than the others. There is also a slight decreasing trend in temperature difference as the pH increases, suggesting that the photothermal performance of NDI-I films decreases at higher pH. In contrast, NDI-F displays the opposite behavior: the photothermal performance of NDI-F films increases as the pH increases. It should be noted that film thickness may influence the absolute photothermal response and should be considered when comparing different film systems.

NDI-I films at pH 6 and NDI-F films at pH 10 generated the highest voltages (Figure 3d), aligning with earlier observations of pH-dependent temperature differences in NDI solutions. The small error in these temperature measurements lends further confidence in the accuracy of the results.

Next, the longevity of the photothermal response was investigated using the Peltier method. The films were irradiated with 617 nm light and the change in potential monitored over time. Visually, the film color faded (Figure 4a) over time due to air oxidation. As the reduced species in the NDI films oxidized back to the ground state, the photothermal performance of the thin films gradually decreased (Figure 4b), suggesting that the photothermal effect is associated with the presence of the reduced states, although thermal effects within the Peltier system during prolonged measurements may also contribute to the observed decrease.

**Figure 4:**
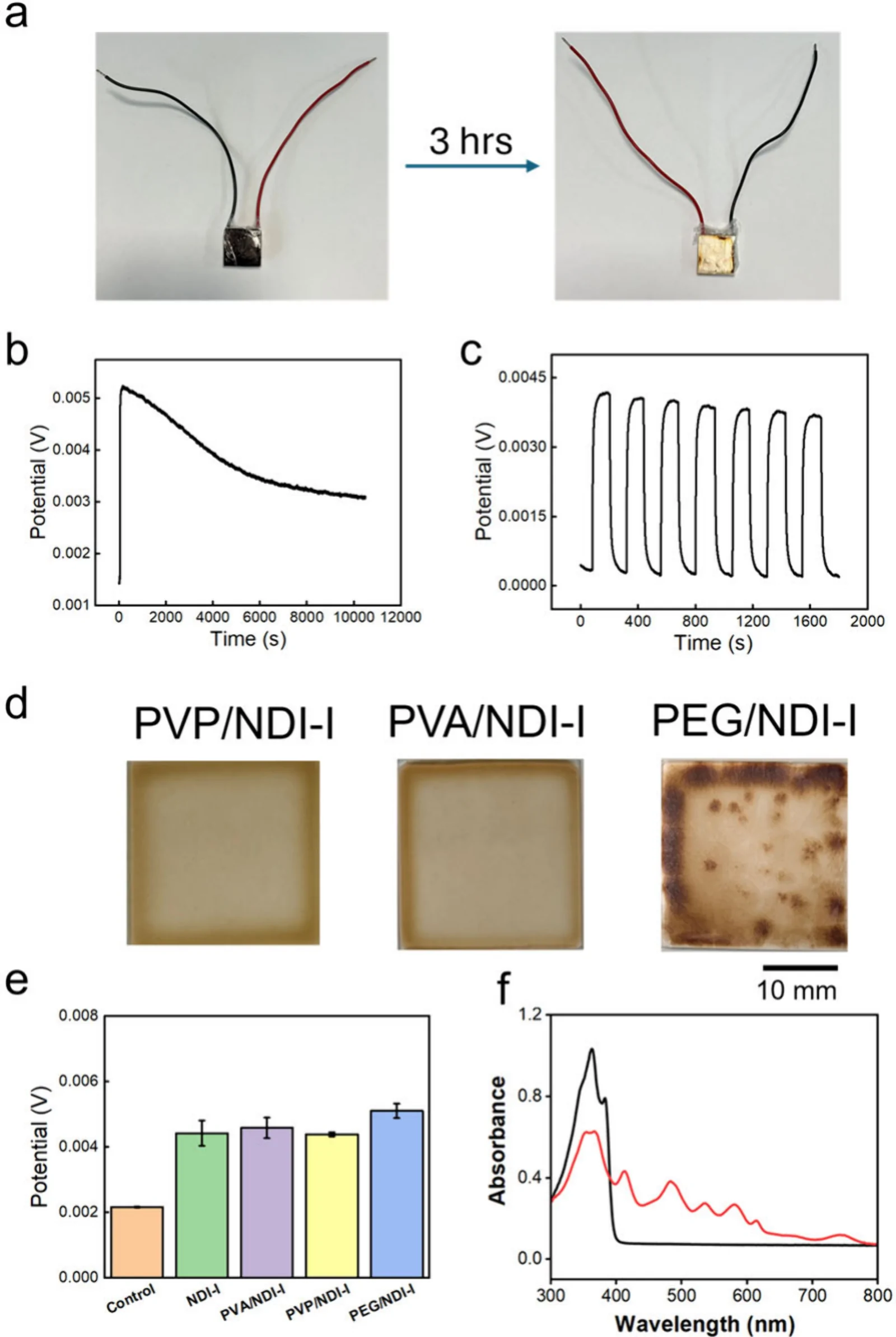
(a) Films attached to the Peltier module after 3 h showing oxidation of the radical anion. (b) Decreased photothermal performance caused by film oxidation after 3 h from oxidation of the radical anion. (c) Voltage response of the Peltier module attached to NDI-I film under on/off cycles of 617 nm irradiation. (d) Thin films prepared from 10 mg/mL NDI-I solutions containing 5% PVP, PVA, or PEG. (e) Voltage output from a Peltier module with or without one side attached to NDI thin films with different polymers. Error bars calculated from standard deviation from three repeats. (f) UV–vis absorption spectra of PVA/NDI-I films before (black) and after (red) 5 min of 365 nm irradiation.

Next, the cyclability of the response was investigated by switching the 617 nm light on and off. This resulted in a rapid response to changes in the generated potential. However, over time, the peak value gradually decreases, which may partly result from the loss of the reduced species, although heat accumulation in the heat sink during prolonged experiments may also contribute to this behavior (Figure 4c).

Because it is the reduced states of NDIs which give rise to their photothermal properties,^35^ the stability of these is key to photothermal efficacy. The incorporation of polymers can impede oxidation through polymer doping or physical prevention of oxidation.^37,38^ Consequently, the oxidation-retarding properties of the polymers PVP (polyvinylpyrrolidone), PVA (poly­(vinyl alcohol)), and PEG (polyethylene glycol) were assessed in our NDI films.

As shown in Figure 4d, PVP/NDI-I and PVA/NDI-I films appeared visually much more homogeneous than the PEG/NDI-I films. The incorporation of polymers into NDI films could lead to changes in the assembly of the NDIs, and therefore changes in performance. This would be apparent in the UV–vis absorption spectra. The hydroxyl groups in PVA can form hydrogen bonds with the imide groups in NDI, altering the electronic environment and causing shifts in absorption peaks. Additionally, PVA can improve the dispersion of NDI molecules, reducing aggregation and resulting in sharper, more defined peaks. Therefore, we investigated whether PVA incorporation affected the peaks generated before and after irradiation. The absorption features of PVA/NDI-I films (Figure 4f) confirm that reduced species are still generated, indicating the photothermal mechanism remains largely unchanged. Compared with the sharp peaks at 350–400 nm in pure NDI-I films, the PVA/NDI-I films display broadened peaks over 300–600 nm after irradiation. This broadening likely results from disrupted π–π stacking by PVA, irradiation-induced structural changes, and additional PVA–NDI interactions. Similar trends were observed in PVA/NDI-F films (Figure S12). Collectively, these effects broaden the absorption profile without altering the reduced-species-based photothermal behavior.

Using the Peltier module setup, we measured the photothermal response of PVP/NDI-I, PEG/NDI-I, PVA/NDI-I, and NDI-I thin films made from pH 8 solutions, and a blank control sample. The results (Figure 4e) indicate that the voltages generated by all films were broadly similar, confirming that the incorporation of the polymer does not impede their photothermal properties. We were able to then investigate the stability and longevity of the photothermal response of films with added polymer. The results show that incorporating PVA is not detrimental to photothermal performance; on the contrary, it might slightly increase the temperature difference, leading to a higher measured potential (Figure 4e). Due to lack of homogeneity of PEG/NDI-I films, we focused exclusively on preparing thin films using the PVA/NDI-I, PVP/NDI-I, and pure NDI-I formulations.

Next, the reduced species’ stability in polymer-blended films was tested. The films were subjected to irradiation with 365 nm light for 10 min. Photographs were taken immediately after irradiation then at various other time points for up to 7 days. The change from a pale yellow to a dark color is indicative of the reduced species.^35^ As shown in Figure 4a, the color of the pure NDI-I films seemed to fade quickly after 3 h, indicating oxidation of the reduced species. We also measured the UV–vis absorption spectrum of each of the NDI-I films after different storage times to monitor the persistence of the reduced species. The results (Figure S13) show that after 3 h, the absorptions at 450 and 800 nm were no longer observed and the material had returned to its original state.

The PVP/NDI-I films exhibited greater longevity, as the film remained dark after 3 h and only began to fade after 2 days. Remarkably, PVA/NDI-I thin films demonstrated the highest stability, remaining dark even after 7 days of storage in ambient conditions (Figure 5a). This enhanced stability is likely related to the presence of the polymer matrix, which provides a more protective environment for the NDI molecules. One possible explanation is that the PVA/glycerol network reduces oxygen diffusion through the film, slowing the oxidation of the NDI radical species. In addition, hydrogen-bonding interactions between the hydroxyl groups of PVA and glycerol and the NDI derivatives may help to stabilize the local molecular environment. Although the exact mechanism cannot be determined from the present data, these effects are consistent with the improved long-term stability observed for the composite films.

**Figure 5:**
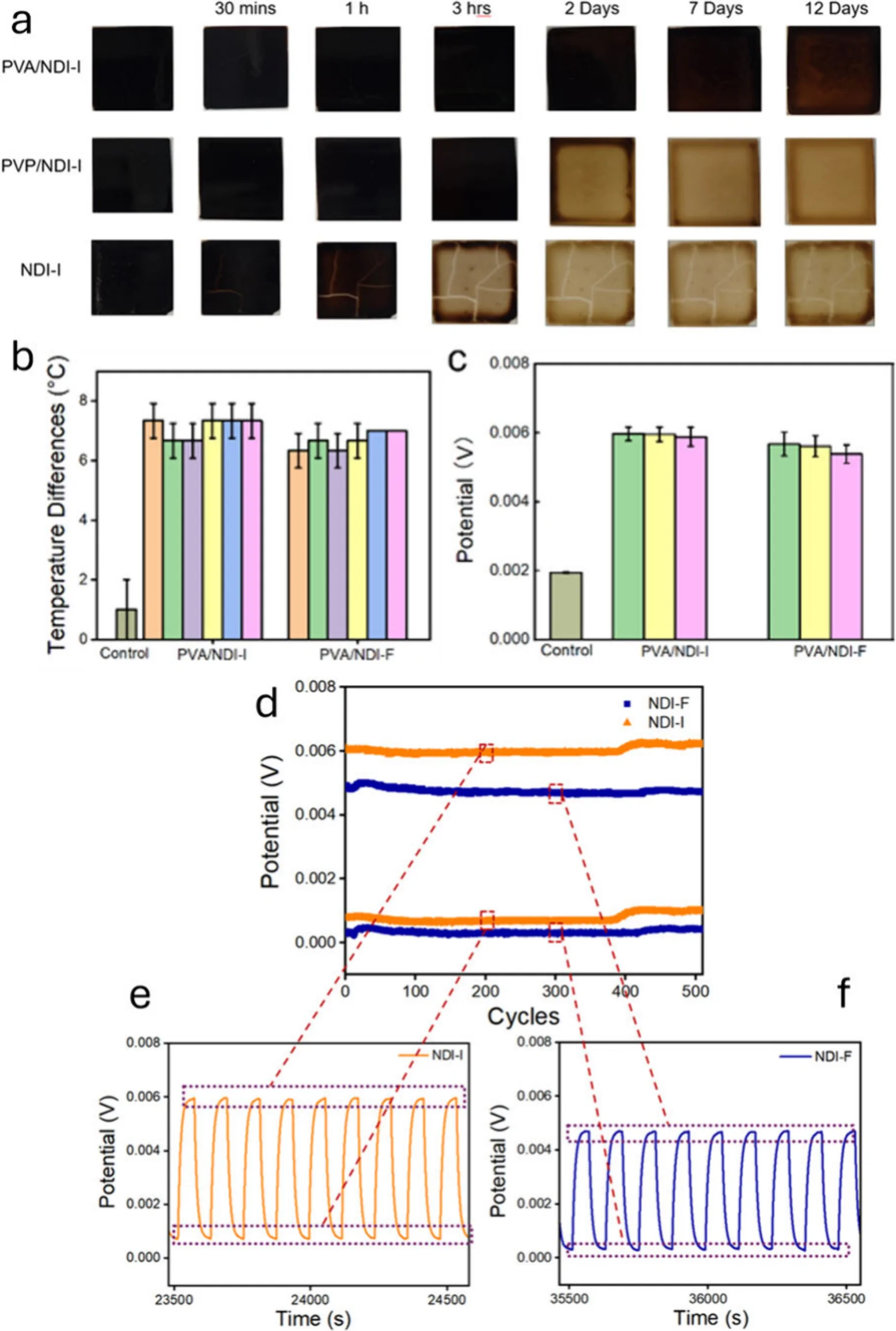
(a) Photographs of PVA/NDI-I, PVP/NDI-I, and pure NDI-I films after irradiation and subsequent storage for varying durations. (b) Temperature rise in PVA/NDI-I and PVA/NDI-F films under irradiation. (c) Voltage output of the Peltier module with PVA/NDI-I or PVA/NDI-F films attached to one side and a heat sink to the other (blue: control; orange: pH 5; green: pH 6; purple: pH 7; yellow: pH 8; gray: pH 9; and pink: pH 10). (d–f) Voltage output from Peltier module under 617 nm light during long-term cycling: (d) shows full cycling data with (e) highlighting cycles 196–204 for PVA/NDI-I (orange), and (f) highlighting cycles 296–304 for PVA/NDI-F (blue). Error bars calculated from standard deviation.

To further assess the reproducibility of the film fabrication process, representative thickness measurements were performed on independently prepared PVA/NDI films. As shown in Figure S14 and Table S2, only small variations in film thickness were observed between replicate samples, demonstrating good consistency in the preparation method.

Using PVA/NDI-I and PVA/NDI-F films, the effect of pH on photothermal performance was investigated. Across the pH range of 5 to 10, the temperature increases measured by thermal camera were very similar (Figure 5b and Figure S15). The PVA/NDI-F films showed slightly lower temperature increases, which is consistent with our previous study indicating that NDI-F exhibits a weaker photothermal response.

Likewise, the potential generated by the Peltier module remained similar across different pH values, consistent with the observed temperature increases (Figure 5c). This behavior may be attributed to PVA preventing oxidation of reduced species within the films, thereby maintaining a high reduced species concentration during irradiation.

The incorporation of PVA improves the resistance to oxidation of the films, as can be judged by observing the color changes. We can use the Peltier module to further support this. We compared 500 cycles for the PVA/NDI-I and PVA/NDI-F films. For each cycle, first the reduced species was generated then the film was irradiated with 617 nm light for 60 s, then allowed to recover for 60 s. The voltage was recorded throughout the process. By comparing the peak and trough values, as well as the differences between them across cycles, we could assess the films’ performance over long time scales. The results show that both PVA/NDI-I and PVA/NDI-F performed well, with the potential generated in each cycle remaining consistent over 500 cycles (Figure 5d–f). Both film types retained a high level of photothermal performance with no significant degradation. NDI-F, while lagging PVA/NDI-I in terms of voltage output, still exhibited very stable cycle-to-cycle temperature increases.

Next, we investigated the mechanical properties of PVA/NDI and PVP/NDI thin films, with and without glycerol, using a nanoindenter (Figure 6a). Mechanical strength is crucial for applications such as flexible electronics, where durability and bendability are required. The results show that the elastic indentation modulus (EIT), reduced modulus (E*), and hardness (HIT) of PVA/NDI films are generally higher than those of PVP/NDI films. However, the addition of 0.5% glycerol caused a dramatic reduction in PVA/NDI modulus (4.44 → 0.06 GPa) and hardness (109.42 → 2.47 MPa), whereas PVP/NDI films were less affected (3.02 → 3.02 GPa; 80.38 → 50.33 MPa). These findings highlight the strong influence of glycerol on PVA/NDI films and demonstrate the potential to tune film mechanics through polymer type and glycerol content for flexible electronics applications. We further investigated the effects of varying PVA and glycerol contents on the mechanical and photothermal properties of PVA/NDI thin films. As shown in Figures S16 and S17, changes in glycerol content significantly affected the hardness, modulus, and viscosity of the films, whereas the overall photothermal performance remained largely unchanged. The film with 5% PVA (Figure S15) exhibited a marginally higher output and higher glycerol contents improved flexibility by lowering the elastic modulus without significantly compromising hardness or photothermal performance.

**Figure 6:**
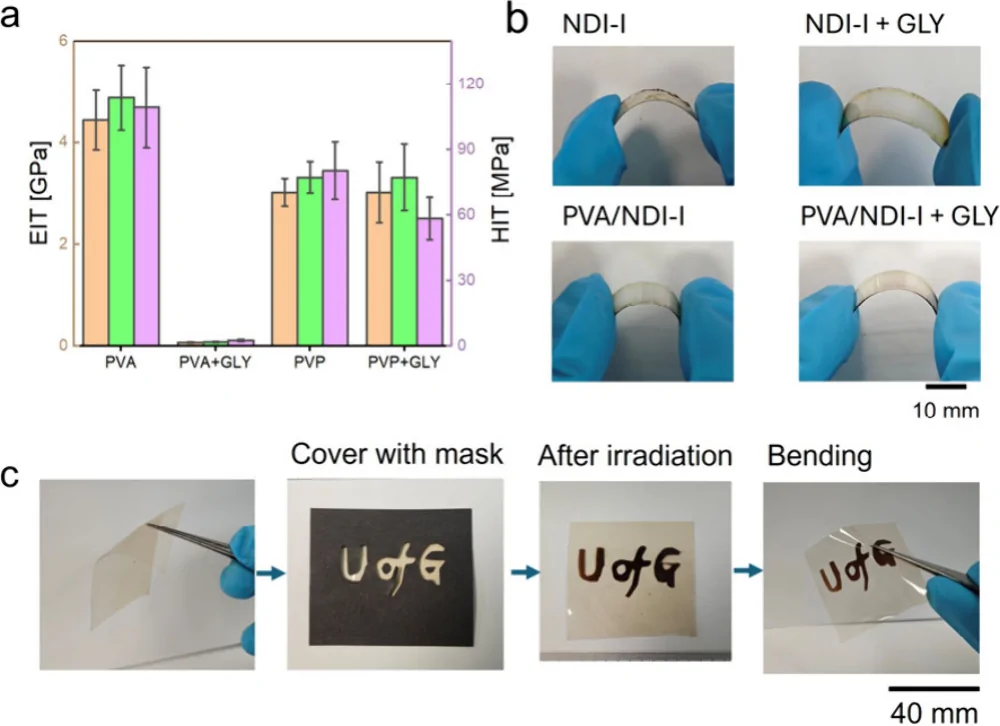
(a) Elastic indentation modulus (orange), reduced elastic modulus (green), and hardness (purple) as a function of indentation depth for PVA/NDI-I and PVP/NDI-I thin films, with and without glycerol. (b) Photographs of flexible films: NDI-I, NDI-I with glycerol, PVA/NDI-I, and PVA/NDI-I with glycerol. (c) Patterned “U of G” lettering and flexibility demonstration of PVA/NDI-I thin films. Error bars calculated from standard deviation.

As the films would be experiencing repeated temperature changes, the thermal stability was also investigated. As shown in Figure S19, thermal stability of NDI-I based films is significantly influenced by the glycerol and PVA contents. In Figure S19a, increasing the glycerol content from 0% to 2% (with fixed 5% PVA) decreases the decomposition onset temperature, indicating a plasticizing effect that reduces thermal resistance. In contrast, Figure S19b demonstrates that increasing the PVA content from 2.5% to 10% (at a fixed glycerol content of 0.5%) leads to a slight improvement in thermal stability, possibly due to enhanced hydrogen bonding and improved film integrity. These results highlight the influence of polymer composition on the thermal behavior and structural robustness of NDI-based composite films.

Finally, the flexibility of the films was investigated. NDI-I, NDI-I with glycerol, NDI-I with PVA, and NDI-I with both PVA and glycerol films were prepared on flexible plastic substrates (see Figure 6b). The edges of the NDI-I films appeared prone to lifting off the substrate, but the addition of PVA and glycerol helped form flexible, robust, and homogeneous films. A mixture of 5% PVA, 10 mg/mL NDI-I, and 1% glycerol was determined to be optimal for preparing large-scale films; the films can be easily peeled from the substrate without distorting their physical properties and the stability of the reduced species. Using these conditions, we prepared 5 cm × 5.5 cm thin films **(** Figure 6 **c)**. These films were then covered with a photomask and irradiated with 365 nm light for 1 min.

After irradiation, a clear dark shape appeared on the films. As seen in the photograph, the pattern did not bleed, suggesting that the reduced species does not diffuse through the film. This is important for applications where the temperature increase must be limited to a targeted area, like in thermal printing or cancer therapy.^1,39^ Additionally, the patterned film demonstrated high flexibility and no damage on bending.

To further assess the suitability of the PVA/ND-I composite films for flexible applications, mechanical bending tests were performed. After 1000 bending–relaxation cycles, the films remained mechanically flexible without visible damage and retained their ability to undergo localized photoinduced color changes under partial irradiation (Figure S20). As the color change arises from the formation of reduced NDI species, the persistence of this behavior indicates that the photothermal functionality of the films is preserved after repeated mechanical deformation. These observations further support the potential of the PVA/NDI composite films for flexible photothermal applications.

## Conclusions

In summary, we successfully introduced glycerol as a plasticizer to fabricate homogeneous and flat photothermal films, as confirmed by microscopic imaging of their microstructures. Photothermal performance was evaluated using two complementary methods: direct temperature mapping under irradiation and voltage generation measurements using a Peltier module. Under 617 nm irradiation at an intensity of 16 mW cm^–2^, the NDI-I films achieved a maximum temperature increase of 7.6 °C (pH 5) and a peak Peltier output voltage of 0.0052 V (pH 6). We would like to note that direct comparison with other materials is extremely difficult due to no standardized reporting or testing of these devices. To further enhance film stability, polymers were incorporated, with poly­(vinyl alcohol) (PVA) identified as the most effective additive for prolonging the lifetime of the reduced species while maintaining reliable photothermal performance. The resulting composite films exhibited rapid and reproducible responses over repeated irradiation cycles, together with good flexibility and ease of patterning into various shapes, highlighting their potential for long-lasting flexible photothermal and wearable electronic applications.

## Supplementary Material



## Notes

Additional experimental details, materials, and methods, including photographs of experimental setup and supplementary figures (PDF)
