# Supplementary material for: Flexible and Long-Lived Naphthalene Diimide-Based Photothermal Films with Rapid and Highly Cyclable Heat Conversion
Source: ACS Appl Polym Mater. 2026 Aug 7;8(16):13642–50. doi: 10.1021/acsapm.6c01376 (PMC13532042; doi:10.1021/acsapm.6c01376)
Supplement: Supplementary file 1 [file ap6c01376_si_001.pdf]

Supporting Information

Flexible and Long-Lived Naphthalene Diimide Based Photothermal Films with Rapid and Highly Cyclable Heat Conversion

Ziyao Xu[a], Bart Dietrich[a], Katarzyna Kozioł[b] and Emily R. Draper[a]*

a. Z. Xu, B. Dietrich and E. R. Draper

School of Chemistry, University of Glasgow, Glasgow, G12 8QQ, UK

b. K. Kozioł

James Watt School of Engineering, University of Glasgow, G12 8QQ, UK

**E-mail:* Emily.Draper@glasgow.ac.uk

Contents

1. Synthetic Procedures S1
   1.1 Materials S1
   1.2 Synthesis of NDIs (NDI-I and NDI-F) S1
   1.3 Preparation of NDI solutions and polymer solutions S1
   1.4 pH measurements S1
   1.5 Film preparation S1
   1.6 Measuring temperature differences using thermal camera S2
   1.7 Measuring temperature differences using Peltier module S2
   1.8 UV-Vis absorption spectroscopy S2
   1.9 Nanoindenter for measuring EIT, E*, and HIT S3
   1.10 Rheometer for viscosity measurement S3
   1.11 Thermogravimetric analysis S3
2 Supplementary Figures S4
   2.1 Effects of solution pH on the preparation of films S4
      2.1.1 NDI films with starting pH of 6 .... S4
      2.1.2 NDI films with starting pH of 8 .... S5
      2.1.3 NDI films with starting pH of 10 .... S5
   2.2 Different methods for preparing photothermal films S6
   2.3 Surface profiles of NDI-I and NDI-I/GLY films S6
   2.4 Photothermal performance of NDI films after irradiation with and without glycerol S7
   2.5 Potential differences generated by Peltier module S7
   2.6 Potential differences generated by Peltier module with NDI films at various pH values attached (without heat sink) S8
   2.7 Film preparation on aluminium foil S9
   2.8 Thermal images of NDI films S9
   2.9 UV-Vis spectra of PVA/NDI-F films before and after irradiation. S9

2.10 UV-Vis monitoring of NDI films during storage S10
2.11 Film Thickness Reproducibility of PVA/NDI-I Films S10
2.12 Thermal images of PVA/NDI films S11
2.13 Mechanical properties of PVA/NDI films with various PVA concentrations..... ....S11
2.14 Mechanical properties of NDI films with various glycerol concentrations .S12
2.15 Potential differences generated by Peltier module with PVA/NDI-I films with different PVA content...S12
2.16 TGA curve of NDI and PVA/NDI films..S13
2.17 Mechanical Durability and Retention of Photothermal Behaviour of PVA/NDI-I Composite Films...S13
3 References...S14

1. Synthetic Procedures

1.1 Materials

Naphthalene-1,4,5,8-tetracarboxylic acid dianhydride (NTCDA), imidazole, and L-isoleucine, were purchased from Fluorochem. L-Phenylalanine was purchased from Merck Life Science, and hydrochloric acid was purchased from Honeywell Fluka. PVA (polyvinyl alcohol, 87-89% hydrolyzed, Mw 31,000-50,000), PVP (polyvinylpyrrolidone, average Mw 40,000), and PEG (polyethylene glycol, average Mw 950-1,050) were purchased from Aldrich. Deionised water was used throughout.

1.2 Synthesis of NDIs (NDI-I and NDI-F)

NDI-I and NDI-F (Figure S1) were synthesised as previously described.[1]

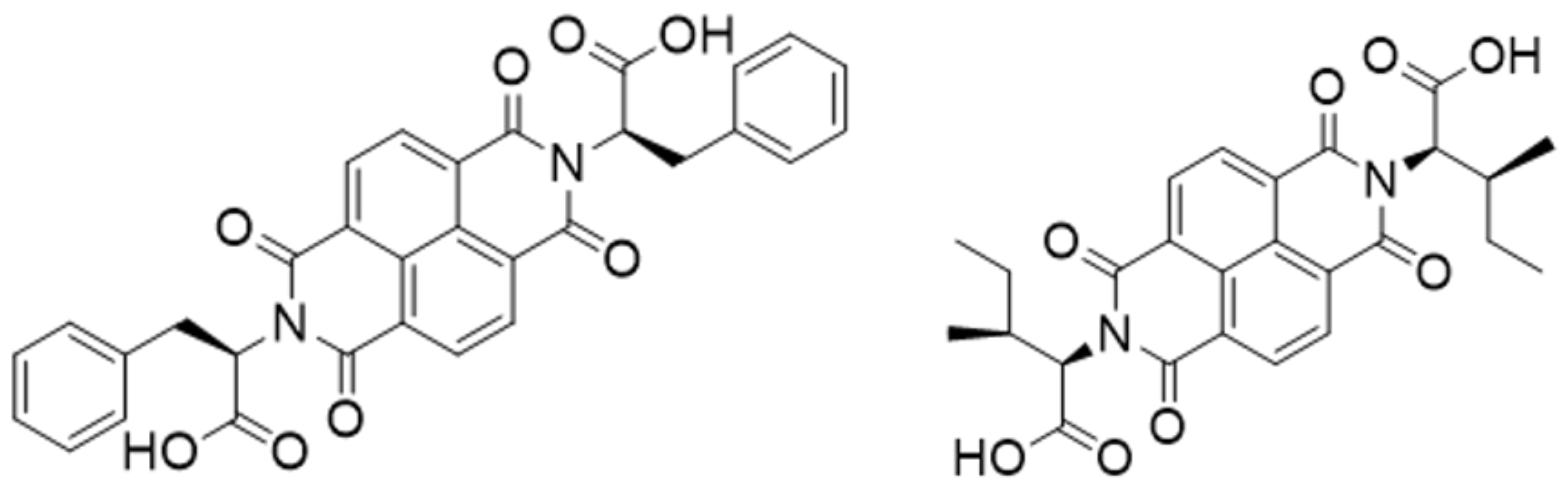

Figure S1. Chemical structures of NDI-F (left) and NDI-I (right).

1.3 Preparation of NDI solutions and polymer solutions

All NDI solutions were prepared by dissolving NDI-I and NDI-F in 20 mL of deionized water to achieve concentrations of 10 mg/mL or 20 mg/mL. To each solution, sodium hydroxide (0.1 M, 2 equivalents, aqueous) was added to achieve dissolution. The mixtures were stirred until complete dissolution of the gelators, typically overnight. The pH of the solutions was then adjusted as necessary by adding either hydrochloric acid (1 M, aqueous) or sodium hydroxide (1 M, aqueous).

The polymers (PVA, PVP, and PEG) were dissolved in water and subjected to stirring in an oil bath at 90°C for 1 hour to obtain 10% weight solutions. These solutions were then mixed in a 1:1 ratio with previously prepared 20 mg/mL NDI solution to obtain 10 mg/mL NDI and 5%wt polymer solutions. Glycerol (typically 0.5%) was then added to the solutions, and the pH was adjusted as appropriate.

1.4 pH measurements

An FC200 pH probe (HANNA instruments) with a 6 mm x 10 mm conical tip was employed for all pH measurements. The probe has a specified precision of ±0.1 pH units.

1.5 Film preparation

Drop-casting:

NDI or PVA/NDI solutions were prepared in advance. Substrates — typically 2.5 cm × 2.5 cm glass, 1.5 cm × 1.5 cm aluminium foil, or Petri dishes — were treated in a plasma cleaner for 5 minutes prior to casting. 0.111 mL/cm^2 of solution was then drop-cast onto the substrate and left to dry overnight. Unless otherwise stated, films prepared in this work were fabricated using

this drop-casting method. NDI solutions used for film preparation contained 10 mg mL^{-1} NDI, while PVA/NDI mixtures typically consisted of 5 wt% PVA and 10 mg mL^{-1} NDI.

Spin-coating:

As PVA/NDI films strongly absorb light, thinner films were required to obtain reliable UV–Vis spectra. Therefore, the films used for UV–Vis measurements were prepared by spin-coating. Spin-coating was carried out at 600 rpm for 30 s, yielding thin PVA/NDI films suitable for optical analysis.

1.6 Measuring temperature differences using thermal camera

Films were prepared by drop-casting 1 mL of the solution into 35 mm diameter Petri dishes and allowing them to dry overnight at room temperature. The dried films were then irradiated with 365 nm light for 5 minutes to generate radical anions. After allowing the samples to cool to room temperature, they were irradiated again with 617 nm light for 5 minutes.

The light intensity was measured using a Thorlabs Optical Power Meter (PM100D) equipped with a Thorlabs sensor (S120VC, 200-1100 nm). During the experiments, the intensity of the 365 nm light was maintained at 80 mW/cm^2 by controlling the distance between the light source and the samples, while the 617 nm light intensity was maintained at 16 mW/cm^2.

For temperature measurement, samples were placed under an inverted box with a cut out hole, allowing the thermal camera lens to focus solely on the sample area. This setup effectively reduced glare, which could otherwise affect temperature accuracy.

Two thermal cameras were used. The thermal images of NDI films were recorded using a FLIR i7 thermal camera, while the PVA/NDI films were recorded using a Seek Thermal Compact Pro camera in conjunction with the Seek Thermal mobile app (Version 2.2.8.0 for iOS). The photothermal performance of the samples was evaluated by comparing their temperature rise above ambient after irradiation.

1.7 Measuring temperature differences using Peltier module

Thin films were prepared by drop-casting 0.25 mL of the solutions onto pieces of aluminium foil measuring 1.5 cm × 1.5 cm. The films were then left to dry at ambient temperature for one day. Once dried, the bare side of the aluminium foil was affixed to one side of a Peltier module using thermal paste to maximise heat transfer. The other side of the Peltier module was attached to a heat sink to dissipate any heat leaking from the hot side, which may reduce the temperature difference across both sides and consequently lower the generated potential.

The films were irradiated, creating a temperature difference between the two sides of the Peltier module, and therefore (*via* the Seebeck effect) a potential difference. This was measured by an Autolab potentiostat in chronopotentiometry mode without applying current. By measuring the voltage generated by the Peltier module, we were able to compare the heat output of each film.

1.8 UV-Vis absorption spectroscopy

Since the drop-cast PVA/NDI films were essentially opaque to our UV-Vis spectrometer, spin-coated films were prepared instead. Glass substrates (2.0 cm × 2.0 cm) were plasma-treated for 5 minutes and immediately transferred to the spin coater. A volume of 0.3 mL of PVA/NDI solution (pH 8) was dispensed onto each substrate, followed by spin-coating at 600 rpm for 30 s. The resulting films were dried at ambient temperature for 2 hours prior to measurement.

UV–Vis absorption spectra were collected using an Agilent Cary 60 UV–Vis spectrophotometer over a wavelength range of 300–800 nm, with a scanning rate of 10 nm s^{-1}.

1.9 Nanoindenter for measuring EIT, E*, and HIT

Nanoindentation measurements were performed using a Step 700 Surface Testing Platform (UNHT3, Anton Paar) with a Berkovich indenter tip. A linear loading profile was applied, reaching a maximum load of 500.00 μN, with loading and unloading rates of 1000 μN/min and a 60-second pause. The constant force was set at 6 μN, with an approach distance of 7500 nm and approach/retract speeds of 5000 nm/min and 2 nm/min, respectively. Each measurement was performed in quadruplicate, using a simple matrix where four points on the film were measured, with a 1 mm gap between each point to prevent surface deformation from a previous indentation affecting subsequent measurements.

The polymer/NDI thin films for nanoindenter measurements were prepared on 2.5 cm x 2.5 cm glass slides *via* drop casting using 0.695 mL of the solution. These films were left to dry at ambient temperature for 1 day. Each measurement comprised of 3 samples and 4 data points per sample.

1.10 Rheometer for viscosity measurement

Viscosity measurements were conducted using an Anton Paar Physica 101 rheometer equipped with a cone plate geometry (50 mm diameter, angle 1°) and a parallel plate measuring system. Each measurement was recorded in triplicate. A volume of 0.65 mL of solution, prepared and pH-adjusted as previously described, was deposited onto the plate and trimmed to fit the geometry. All experiments were conducted at a constant temperature of 25°C, maintained using a water bath.

1.11 Thermogravimetric analysis

Thermogravimetric analysis (TGA) was conducted using a TA Instruments SDT Q600 at the University of Glasgow. Measurements were carried out from room temperature to 800 °C at a heating rate of 10 °C per minute, using a platinum pan with a 100 μL capacity.

1.12 Bending Durability Test

Large films were prepared by drop-casting the solutions at a loading of 0.111 mL cm^{-2}. The films were dried in a fume hood for 24 h and then cut into strips measuring 7 cm × 1 cm. For the bending test, the stage was displaced by 3.5 cm from its initial position and then immediately returned, with no dwell time between cycles. Each bending–relaxation cycle was completed in 16 s, and the samples were subjected to 1,000 consecutive cycles.

2 Supplementary Figures

2.1 Effects of solution pH on the preparation of films

2.1.1 NDI films with starting pH of 6

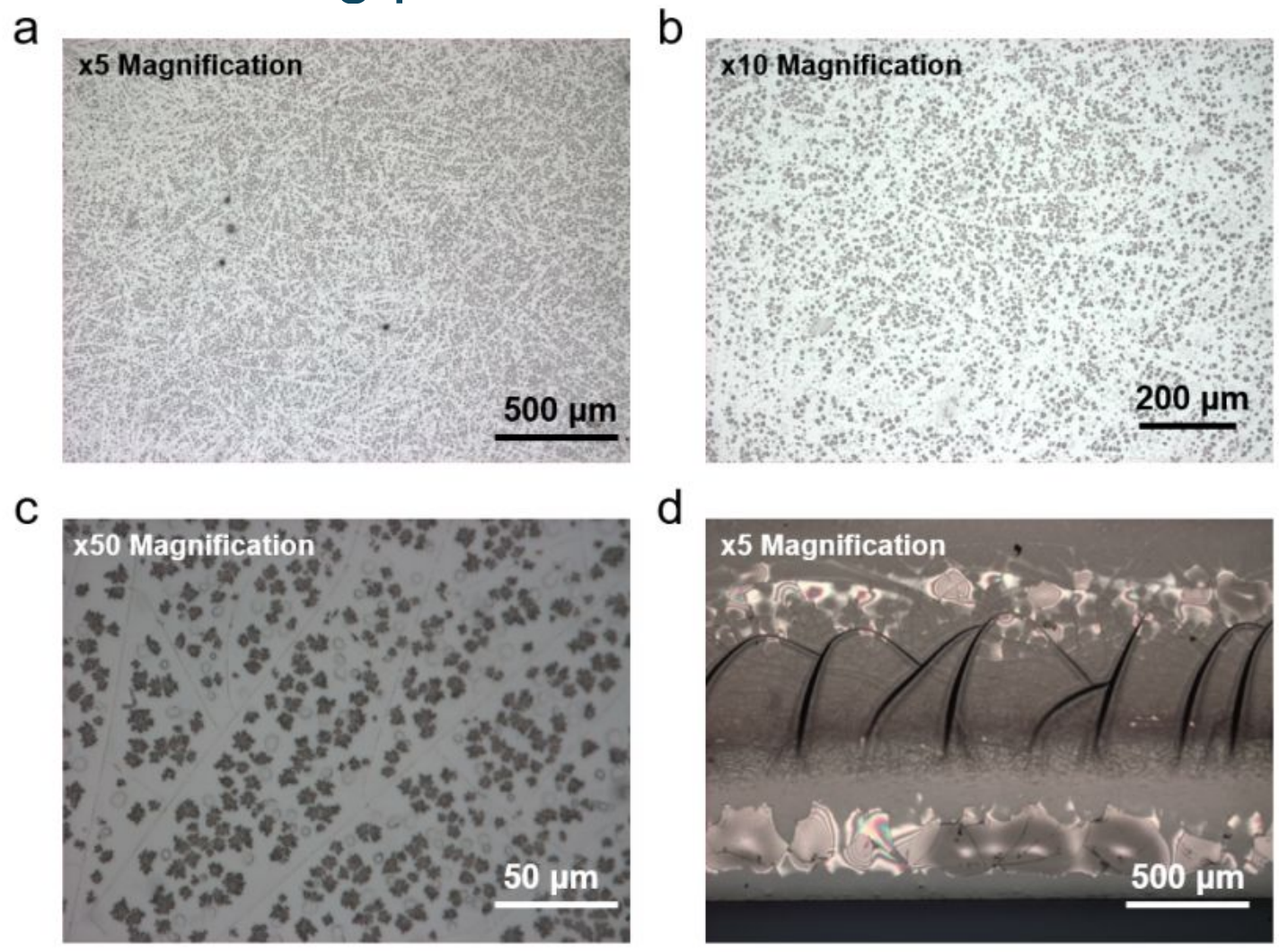

Figure S2. Microscopic images of the middle region of NDI-I films prepared from pH 6 solutions at (a) 5×, (b) 10×, and (c) 50× magnification. (d) Microscopic image of the edge region of the same film at 5× magnification.

2.1.2 NDI films with starting pH of 8

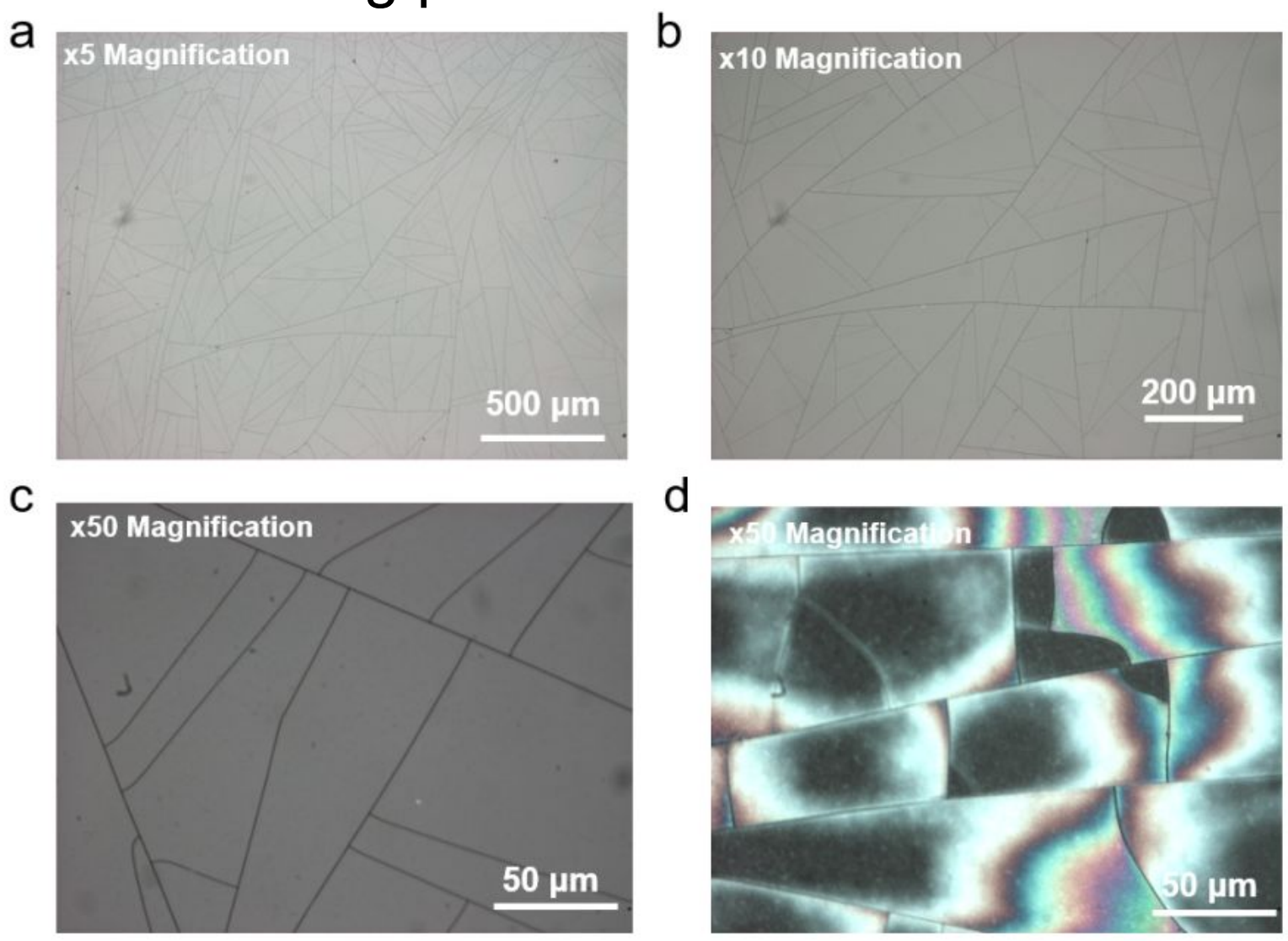

Figure S3. Microscopic images of the middle region of NDI-I films prepared from pH 8 solutions at (a) 5×, (b) 10×, and (c) 50× magnification. (d) Microscopic image of the edge region of the same film at 5× magnification.

2.1.3 NDI films with starting pH of 10

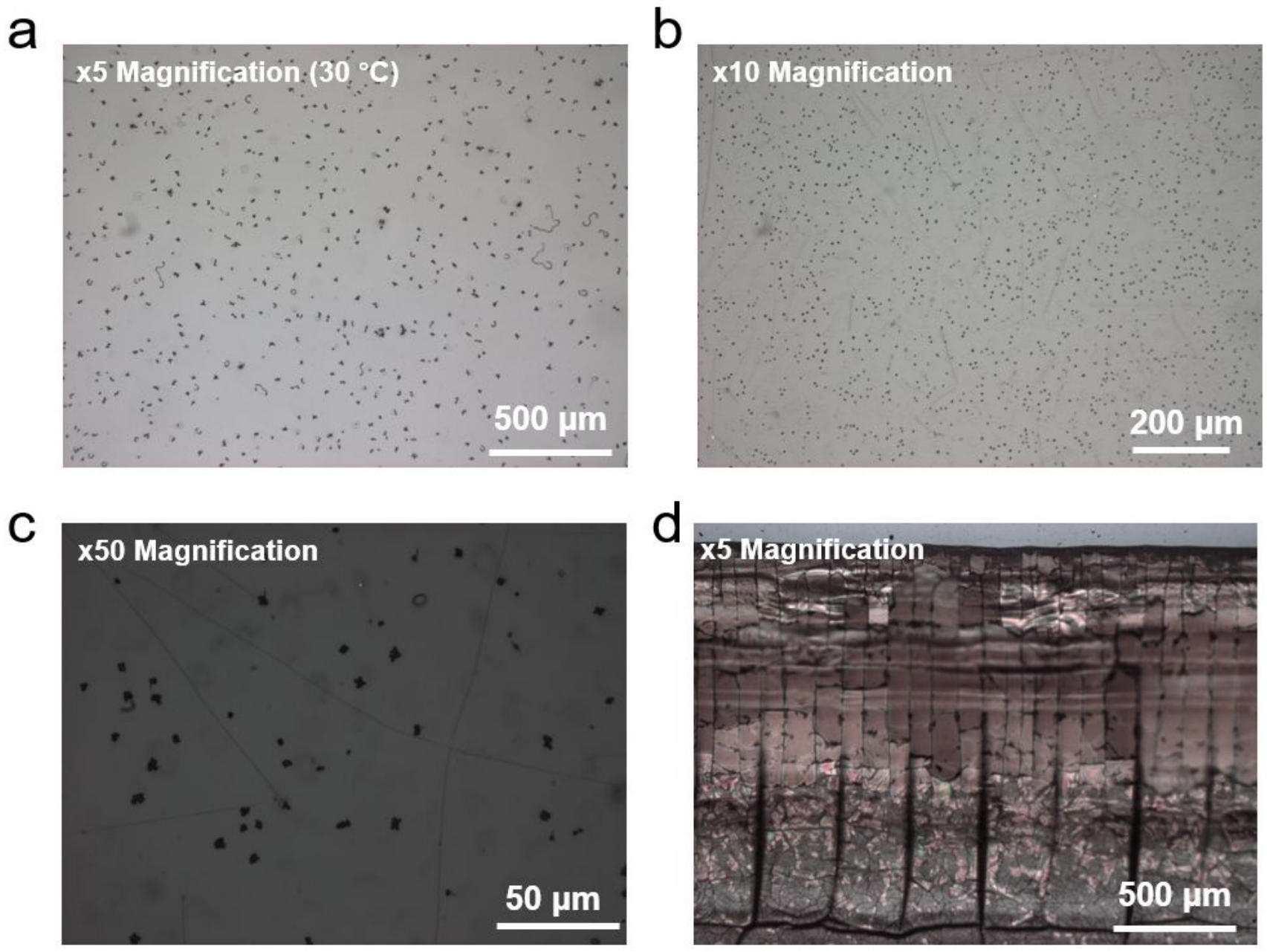

Figure S4. Microscopic images of the central region of NDI-I films prepared from pH 10 solutions at 30 °C: (a) 5×, (b) 10×, and (c) 50× magnification; (d) the edge region of the same film at 5× magnification.

2.2 Different methods for preparing photothermal films

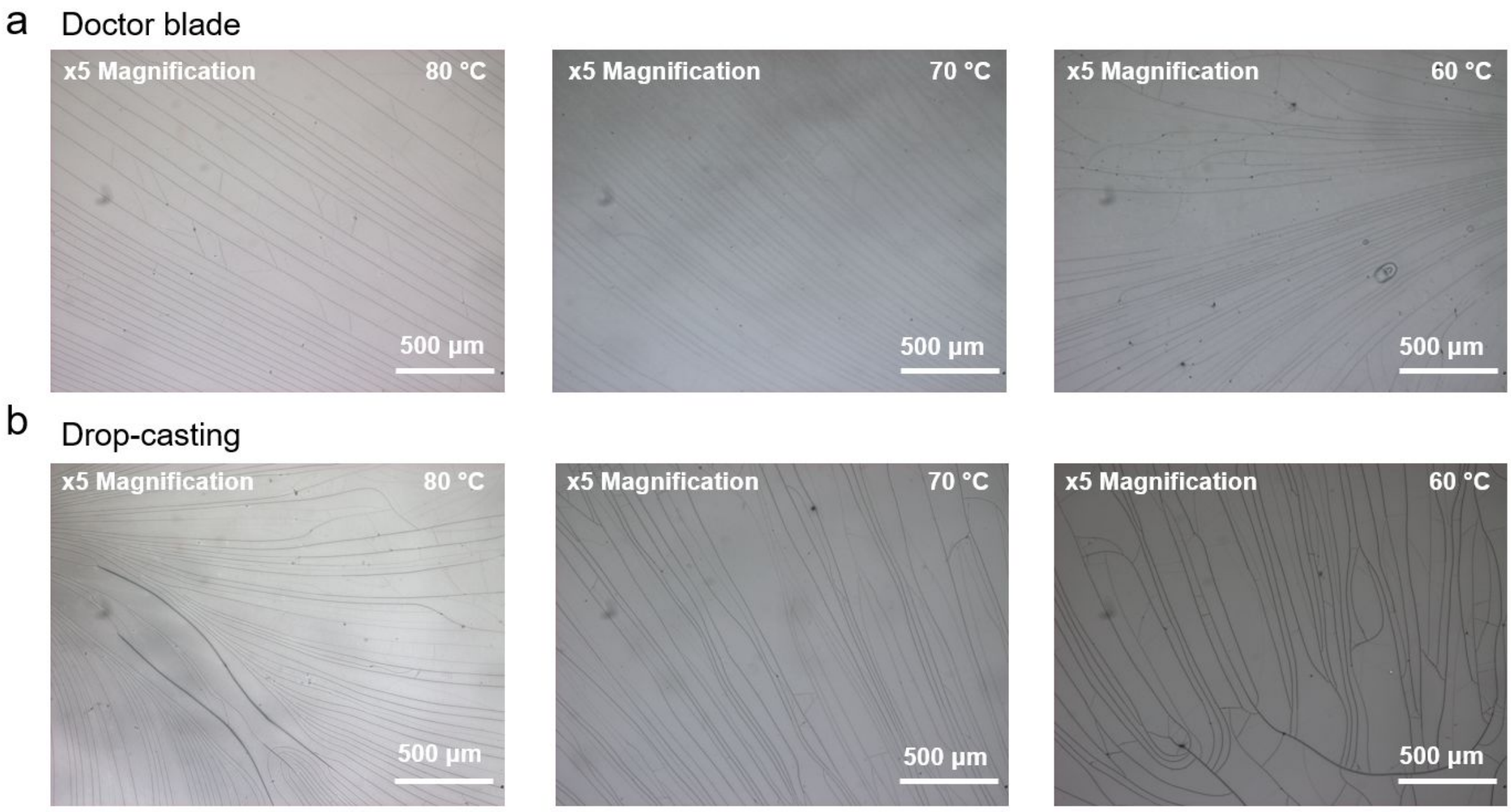

Figure S5. Microscopic images of the middle region of NDI-I films prepared by (a) doctor blading and (b) drop-casting methods, annealed at 60 °C, 70 °C, and 80 °C.

2.3 Surface profiles of NDI-I and NDI-I/GLY films.

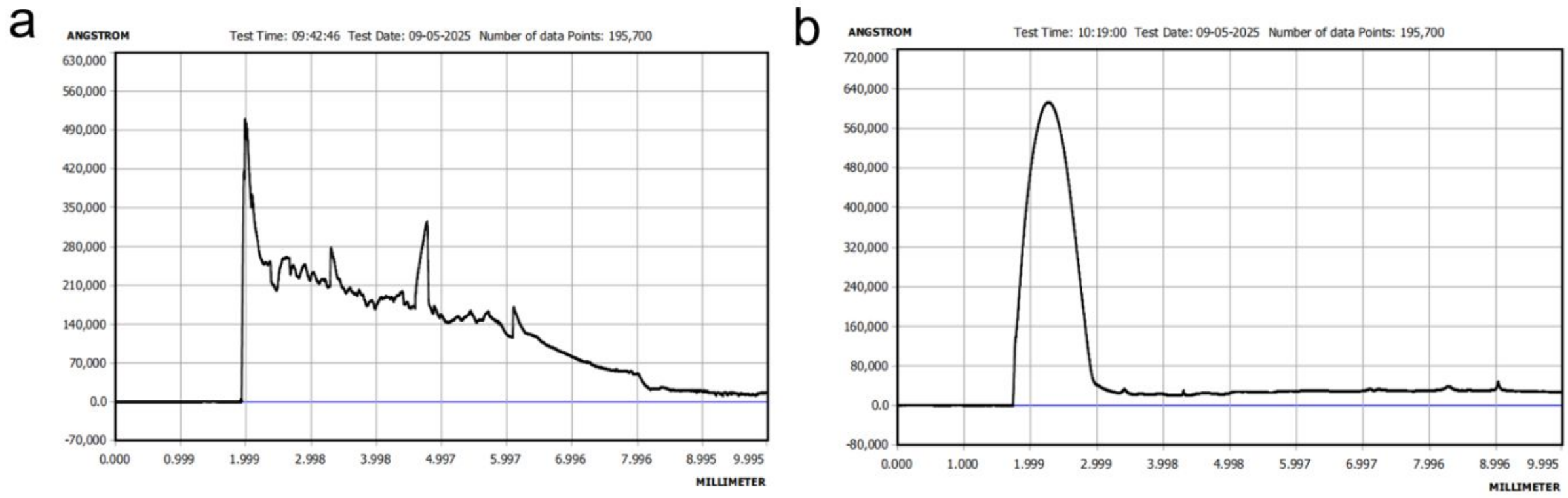

Figure S6. Surface profiles of (a) NDI-I film and (b) NDI-I/GLY film. The scan starts from the substrate region (left), followed by the coffee-ring ridge, and then the film surface.

2.4 Photothermal performance of NDI films after irradiation with and without glycerol

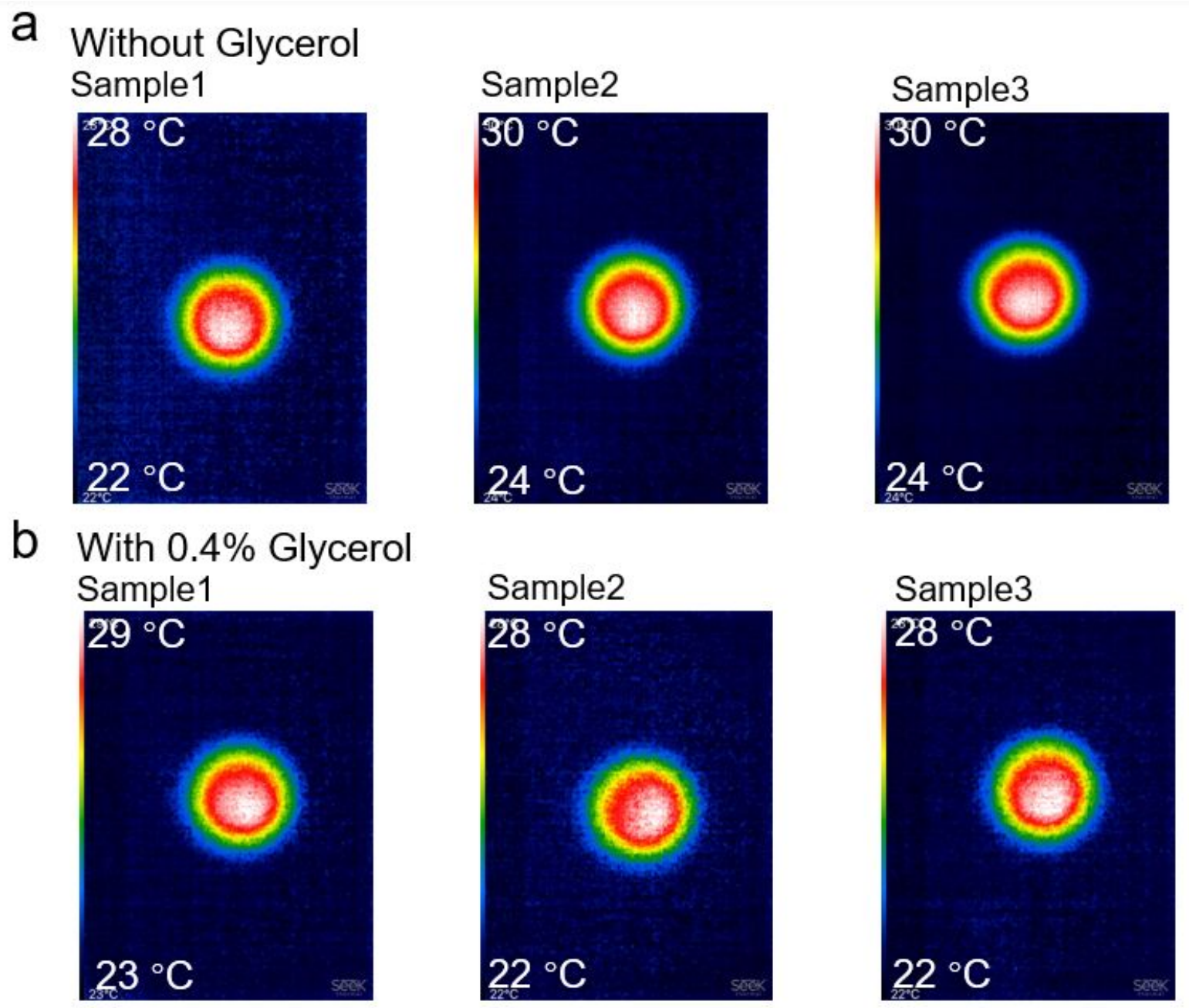

Figure S7. Photothermal images of NDI-I solutions captured by a thermal camera without (a) and with (b) added glycerol. The temperatures displayed at the top and bottom of each image represent the maximum and minimum values of the automatically adjusted temperature scale of the thermal camera.

2.5 Potential differences generated by Peltier module

Table S1. Portion of the raw data for the NDI-I sample recorded prior to irradiation.

Time (s)	WE(1).Potential (V)
0.677310998	0.000518799
0.777310998	0.000521851
0.877310998	0.000518799
0.977310998	0.000524902

1.077310997	0.000521851
1.177310997	0.000521851
1.277310997	0.000521851
1.377310997	0.000521851
1.477310996	0.000524902

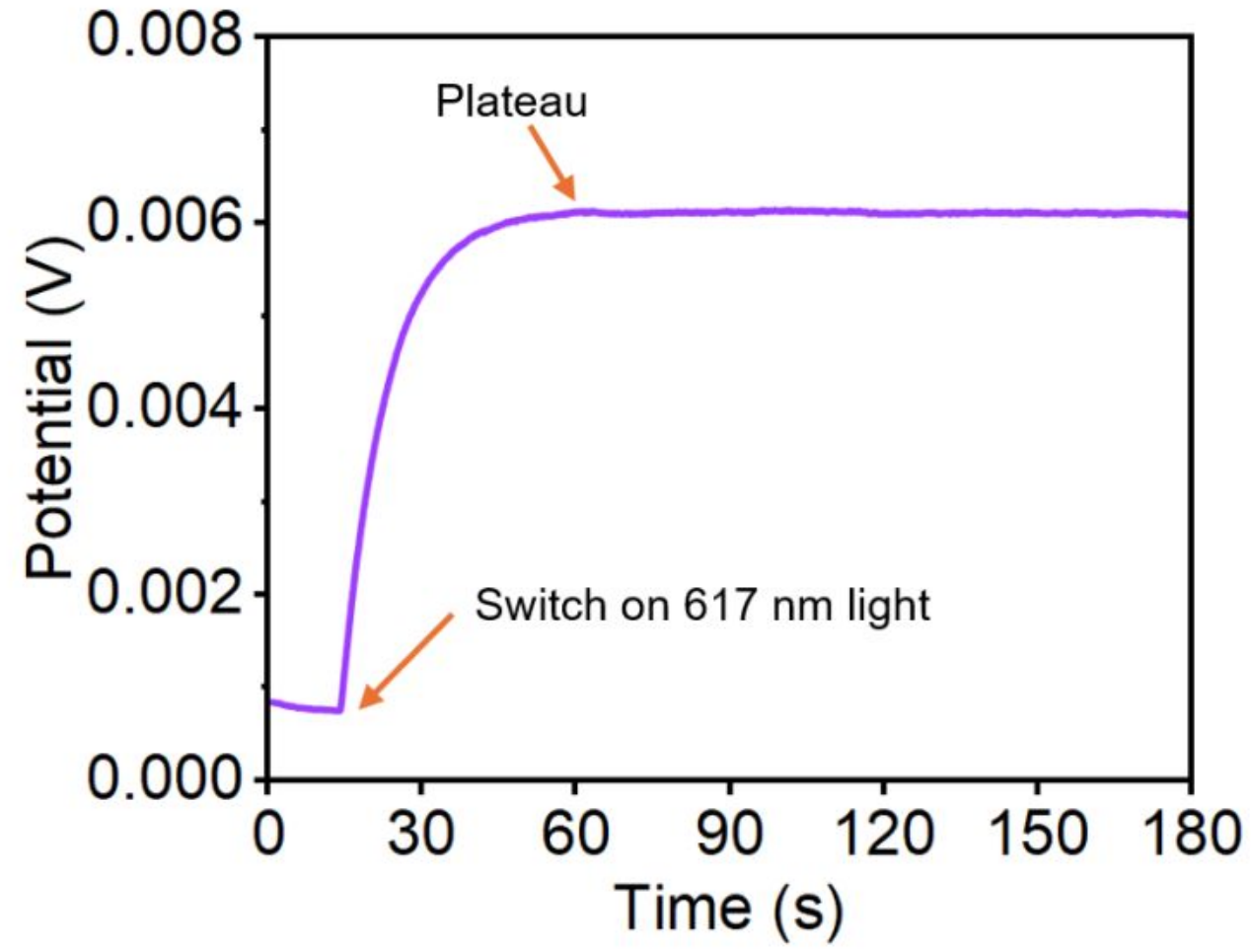

Figure S8. Potential difference generated by Peltier module with one side attached to NDI film under 617 nm light irradiation.

2.6 Potential differences generated by Peltier module with NDI films at various pH values attached (without heat sink)

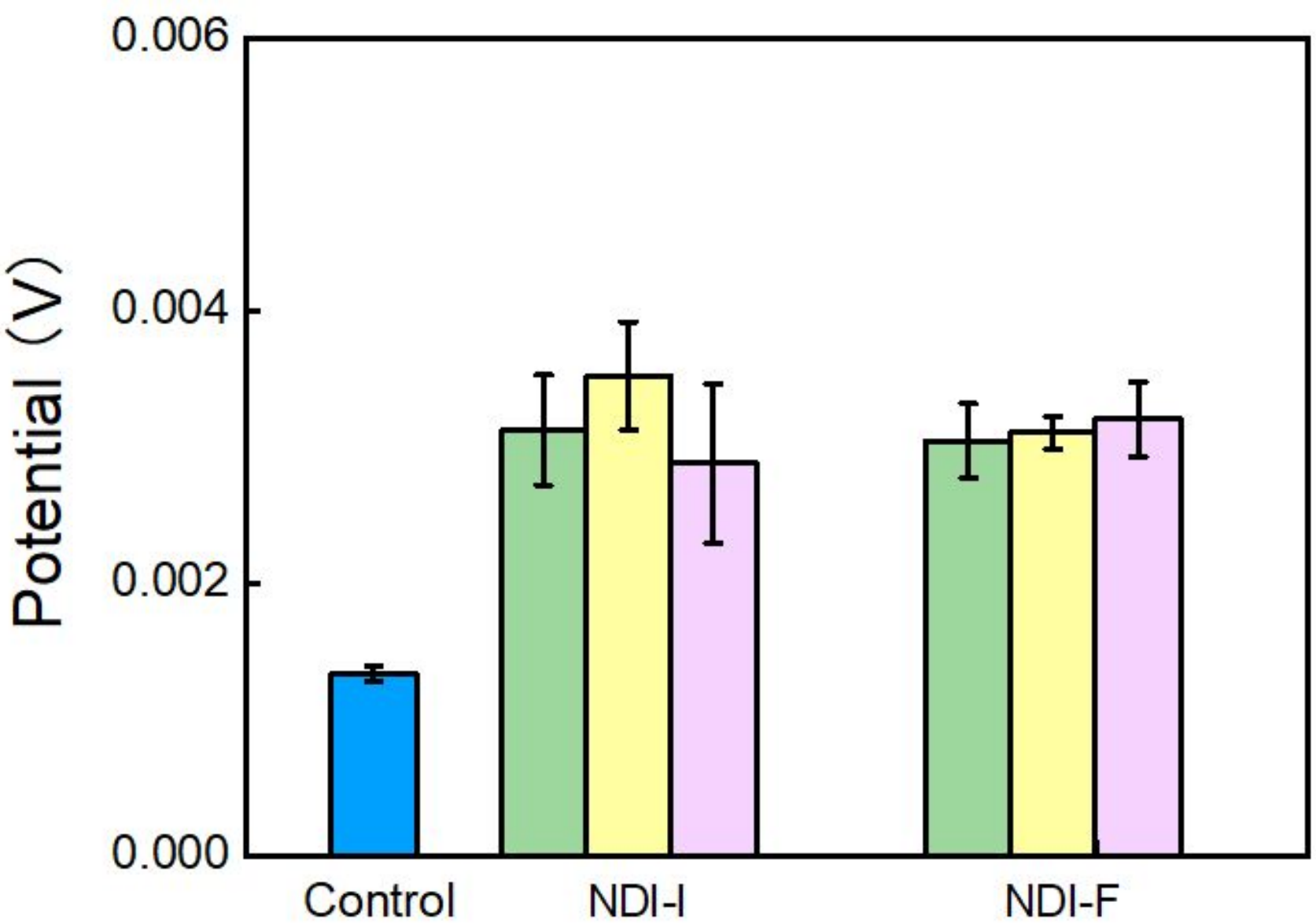

Figure S9. Potentials generated by Peltier module with one side attached to NDI films prepared at different pH values, without a heat sink, under 617 nm light irradiation. Blue bar represents the control sample; green: pH 6; yellow: pH 8; and pink: pH 10. Error bars calculated from standard deviation.

2.7 Film preparation on aluminium foil

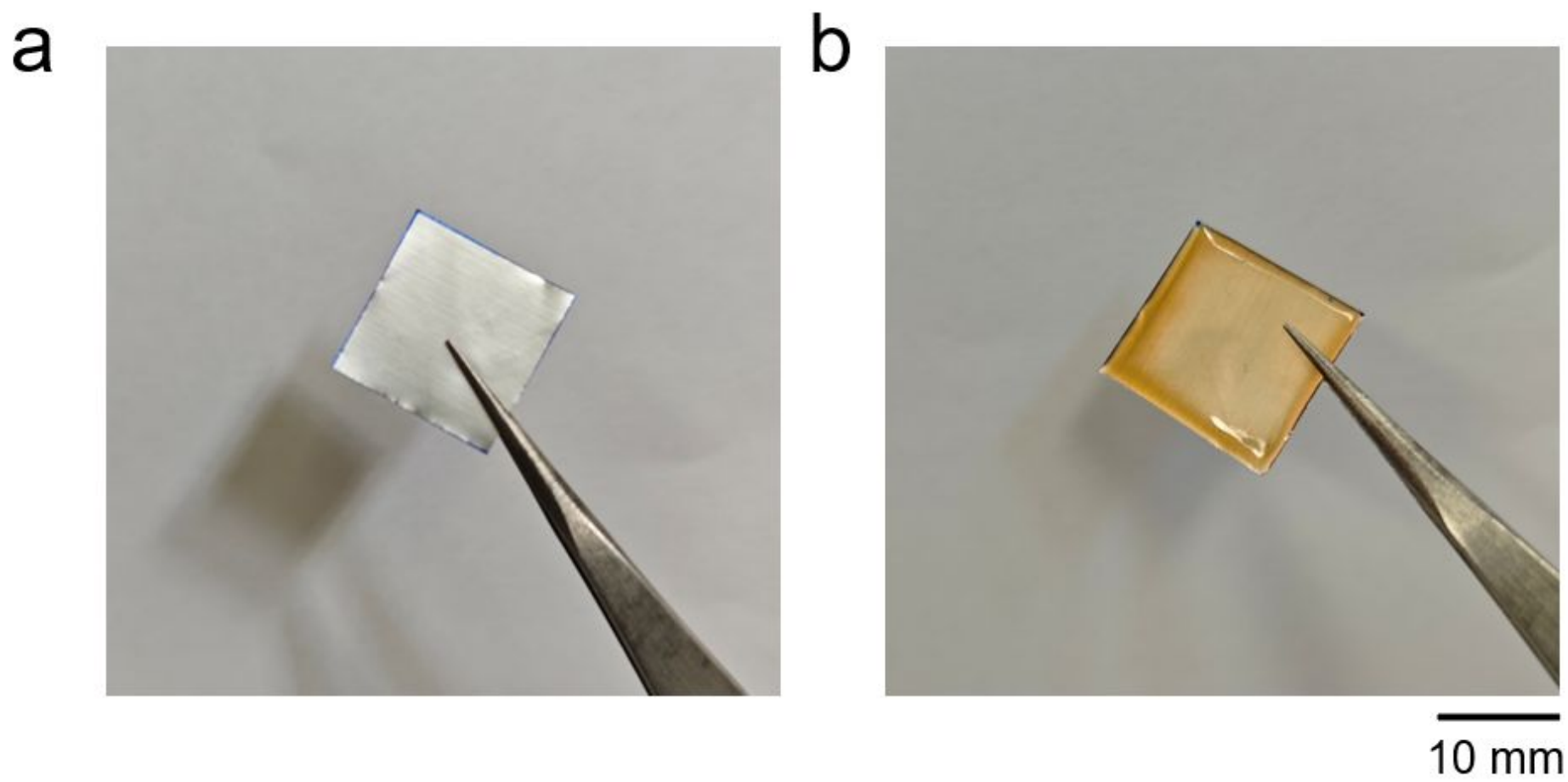

Figure S10. (a) Cut aluminium foil (1.5 × 1.5 cm). (b) NDI-I film with glycerol prepared on aluminium foil at pH 8.

2.8 Thermal images of NDI films

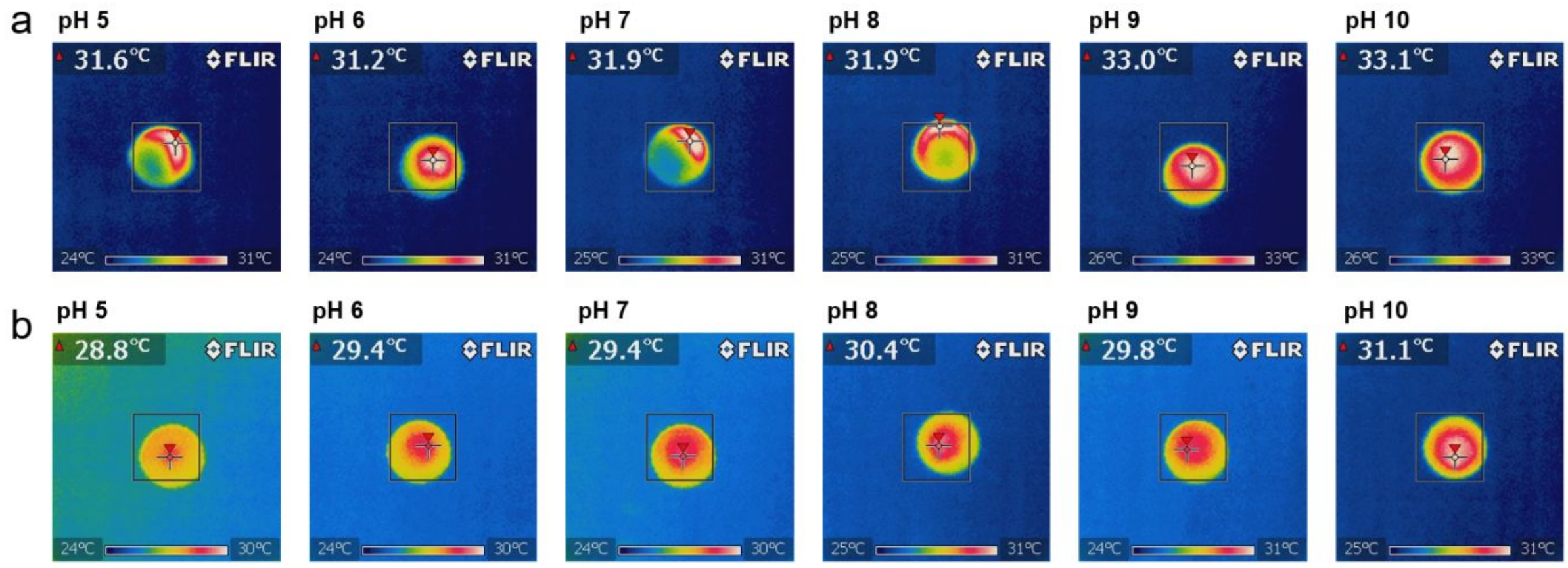

Figure S11. Representative thermal images of (a) NDI-I and (b) NDI-F thin films prepared at pH from 5 to 10. The images were recorded immediately after 5 minutes of 617 nm irradiation, following an initial 5-minute 365 nm irradiation and a subsequent cooling phase.

2.9 UV-Vis spectra of PVA/NDI-F films before and after irradiation.

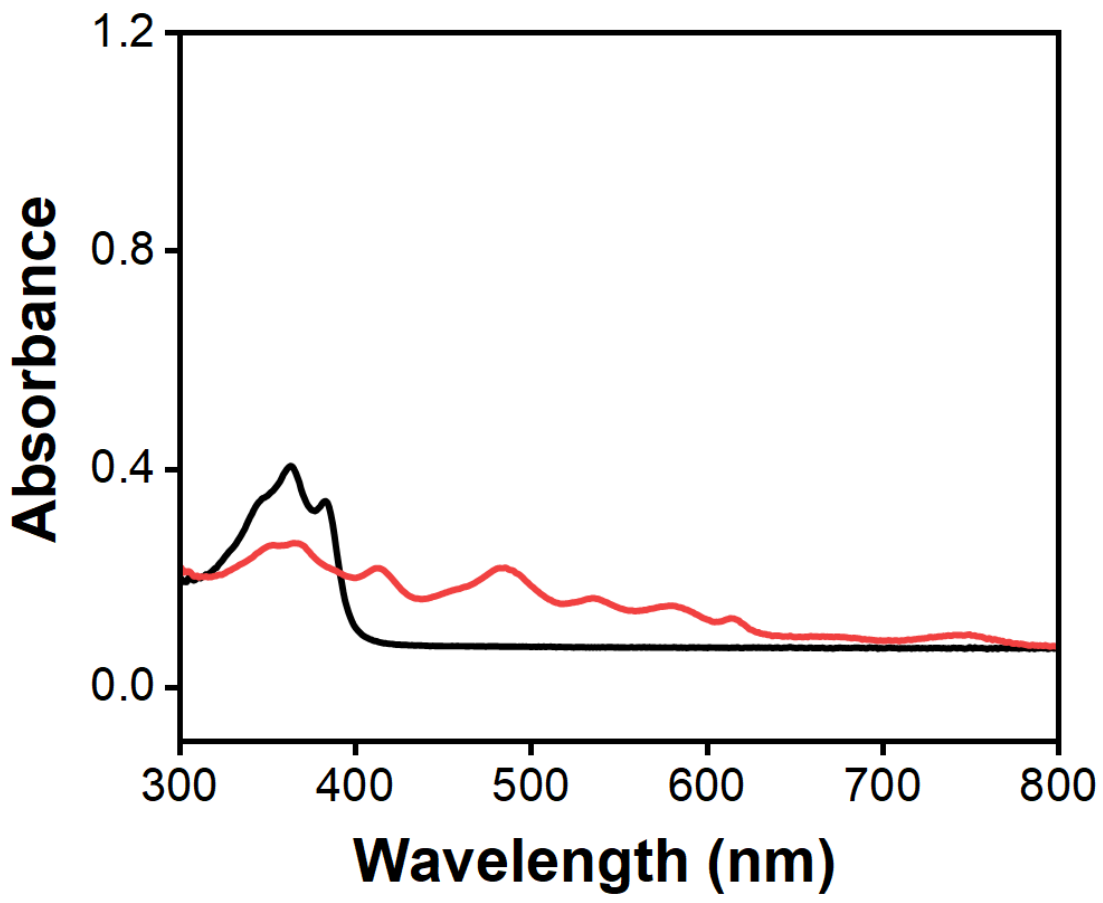

Figure S12. UV–Vis spectra of PVA/NDI-F films before (black) and after (red) irradiation.

2.10 UV-Vis monitoring of NDI films during storage

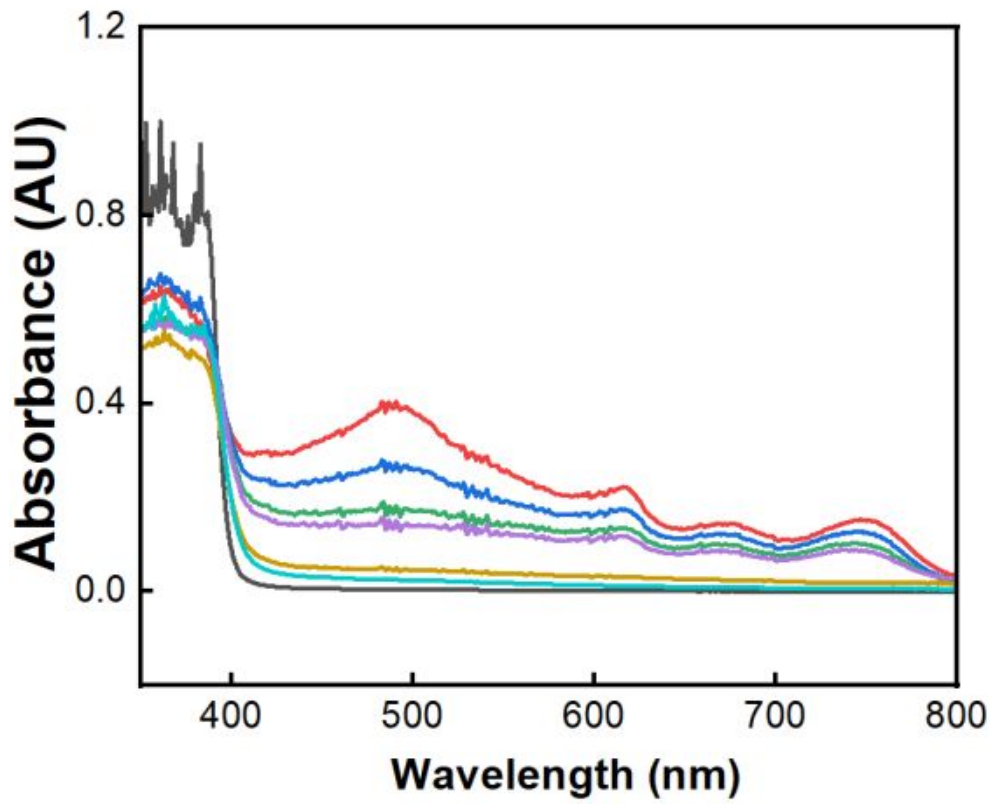

Figure S13. UV-vis absorption spectra of NDI-I film after 365 nm light irradiation, followed by storage in a dark ambient environment for various durations. Black: original; red: immediately after irradiation; blue: 10 min; green: 30 min; purple: 1 h; mustard yellow: 3 h; cyan: 1 day.

2.11 Film Thickness Reproducibility of PVA/NDI-I Films

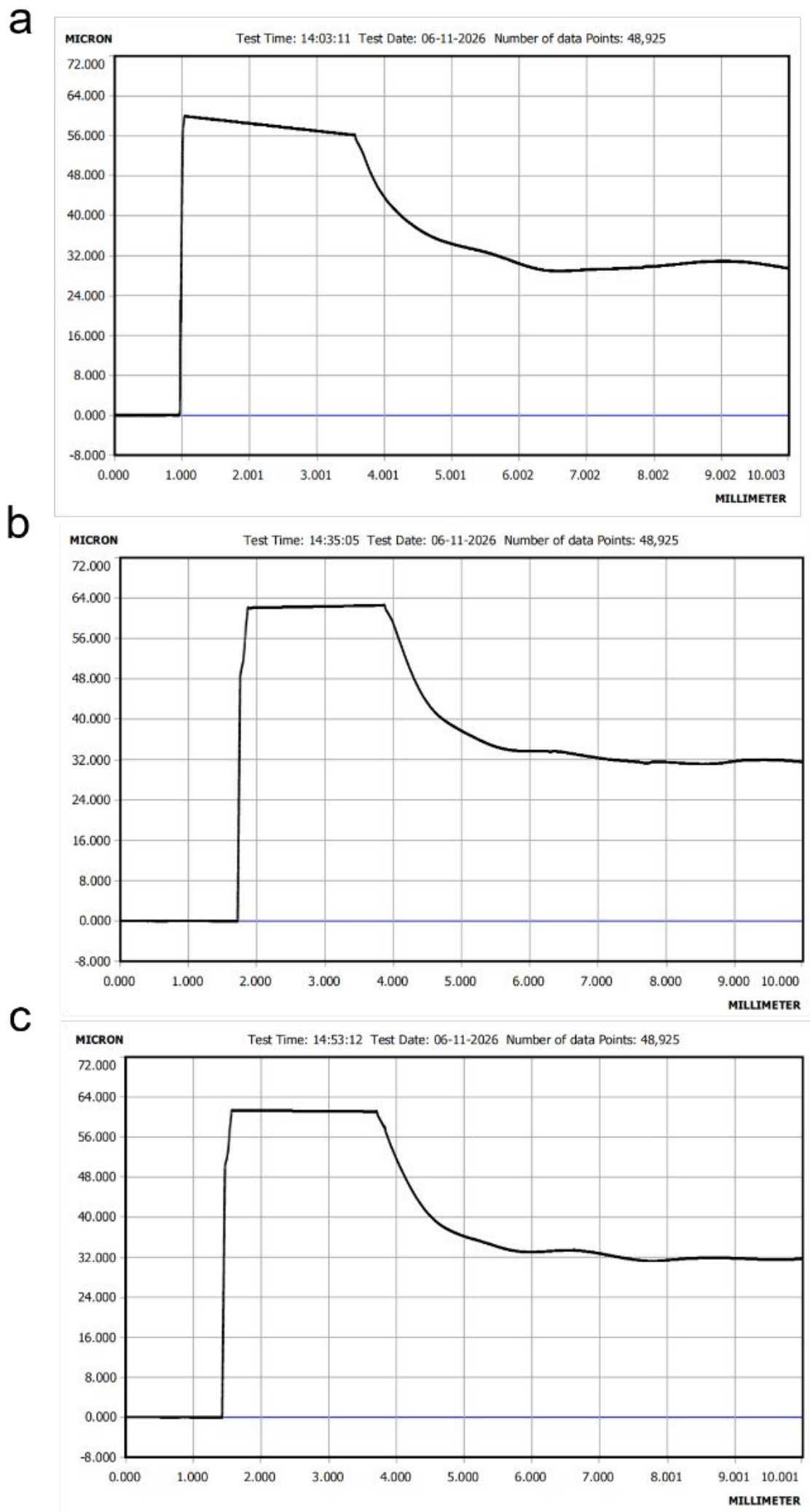

Figure S14. Representative thickness profiles from three independently prepared PVA/NDI-I films prepared at pH 5, with corresponding thickness measurements at three positions on each film, demonstrating the reproducibility of the film fabrication process.

Table S2. Thickness measurements at three locations for each of three independently prepared PVA/NDI-I films prepared at pH 5.

Thickness (µm)	Measurement 1	Measurement 2	Measurement 3
Sample 1	29.94	30.66	32.97
Sample 2	32.39	34.30	32.07
Sample 3	31.34	31.99	32.85

2.12 Thermal images of PVA/NDI films

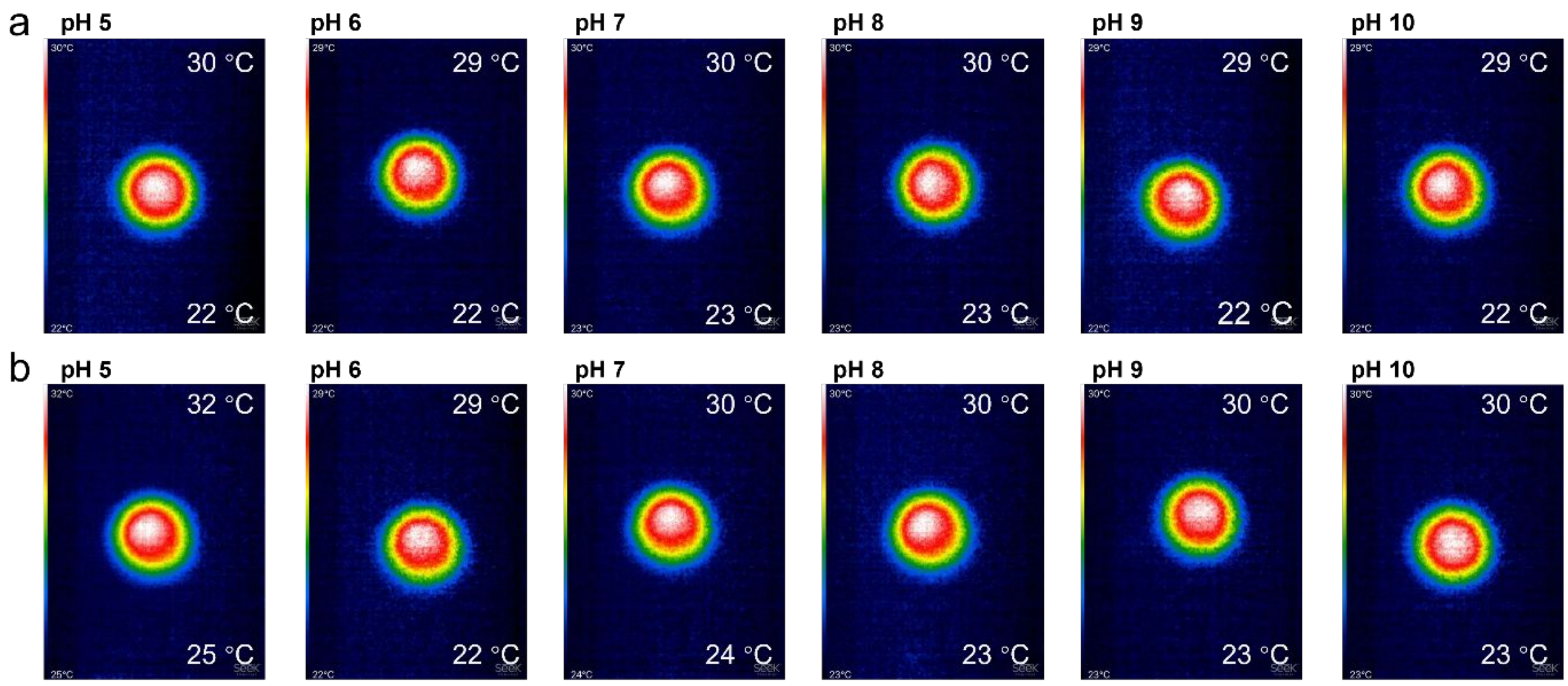

Figure S15. Representative thermal images of (a) PVA/NDI-I and (b) PVA/NDI-F thin films prepared at pH from 5 to 10. The images were recorded immediately after 5 minutes of 617 nm irradiation, following an initial 5-minute 365 nm irradiation and a subsequent cooling phase. The temperatures displayed at the top and bottom of each image represent the maximum and minimum values of the automatically adjusted temperature scale of the thermal camera.

2.13 Mechanical properties of PVA/NDI films with various PVA concentrations

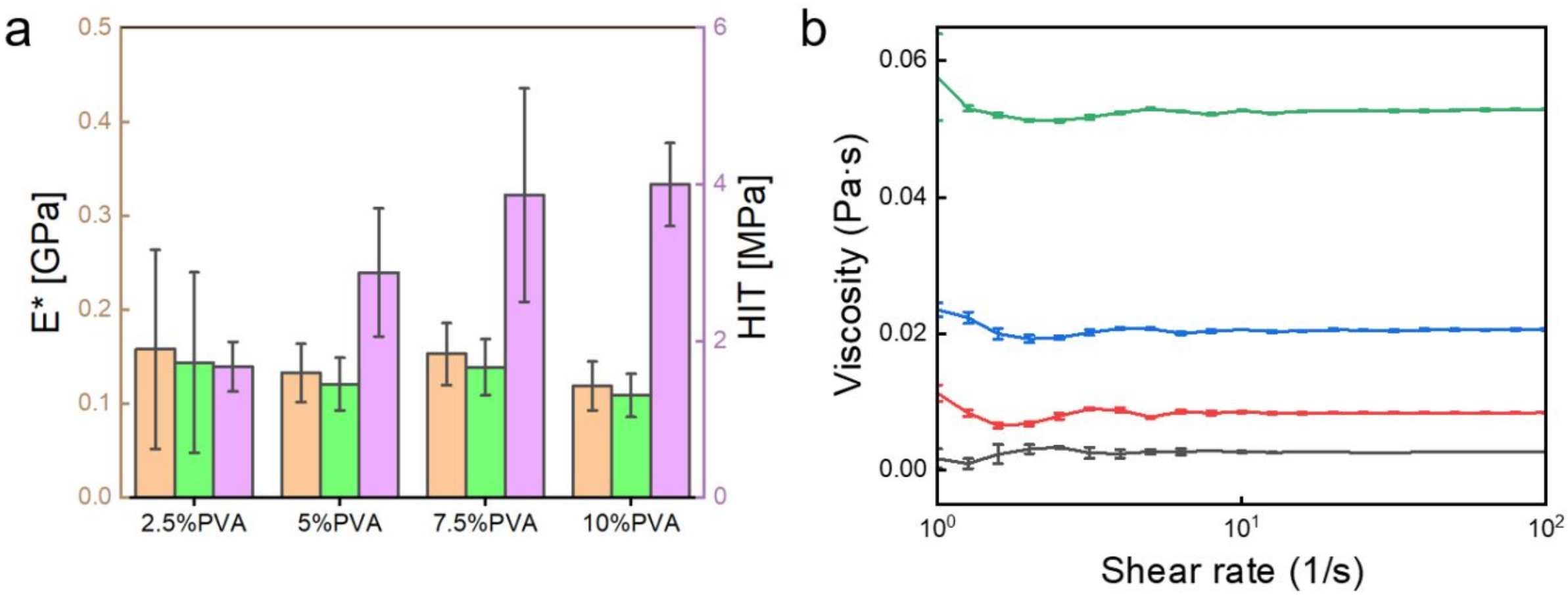

Figure S16. (a) Elastic indentation modulus (orange), reduced elastic modulus (green), and Hardness at Indentation Depth (purple) of PVA/NDI-I thin films with varied PVA content. (b)

The viscosity of solutions used for preparing thin films with varied PVA content. Black line: 2.5% PVA, red: 5% PVA, blue: 7.5% PVA, and green: 10% PVA. Error bars calculated from standard deviation.

2.14 Mechanical properties of NDI films with various glycerol concentrations

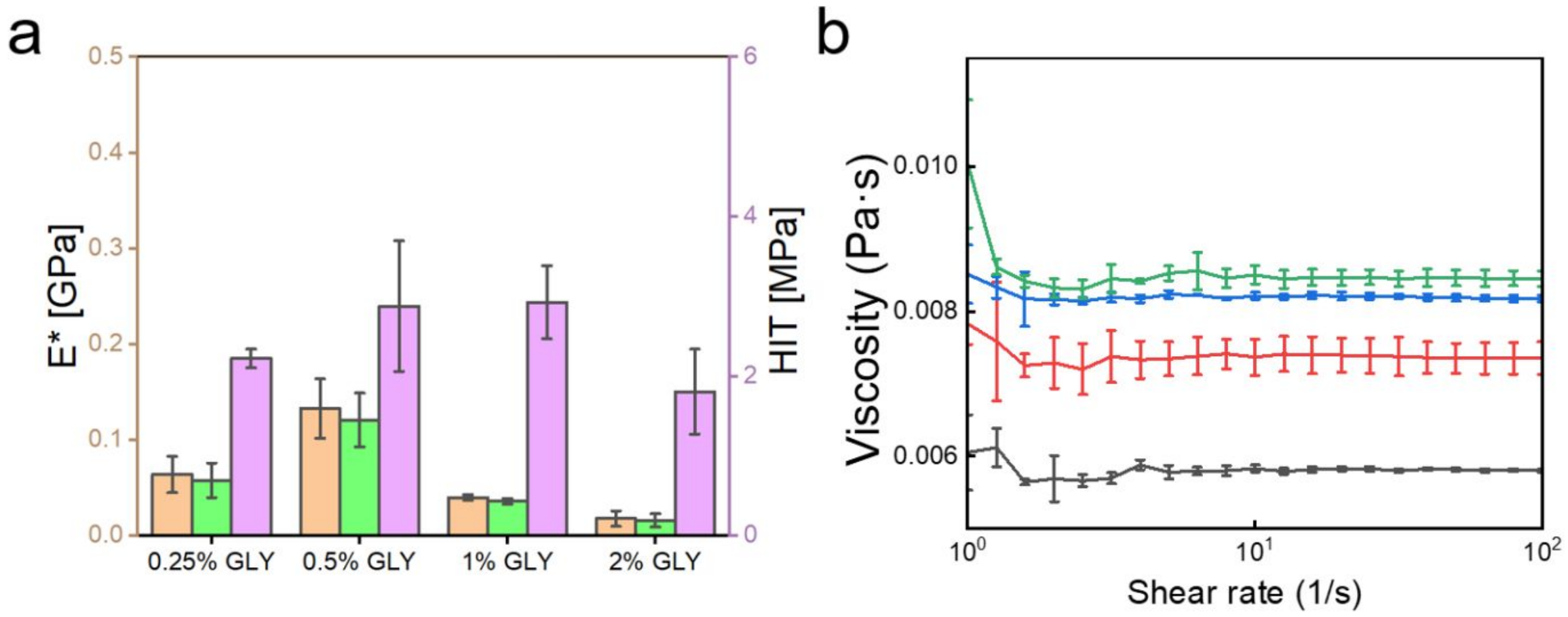

Figure S17. (a) Elastic indentation modulus (orange), reduced elastic modulus (green), and hardness at indentation depth (purple) of PVA/NDI-I thin films with varying glycerol content. (b) Viscosity of the corresponding precursor solutions. Black: 0.25 wt% glycerol; red: 0.5 wt% glycerol; blue: 1 wt% glycerol; green: 2 wt% glycerol. Error bars calculated from standard deviation.

2.15 Potential differences generated by Peltier module with PVA/NDI-I films with different PVA content

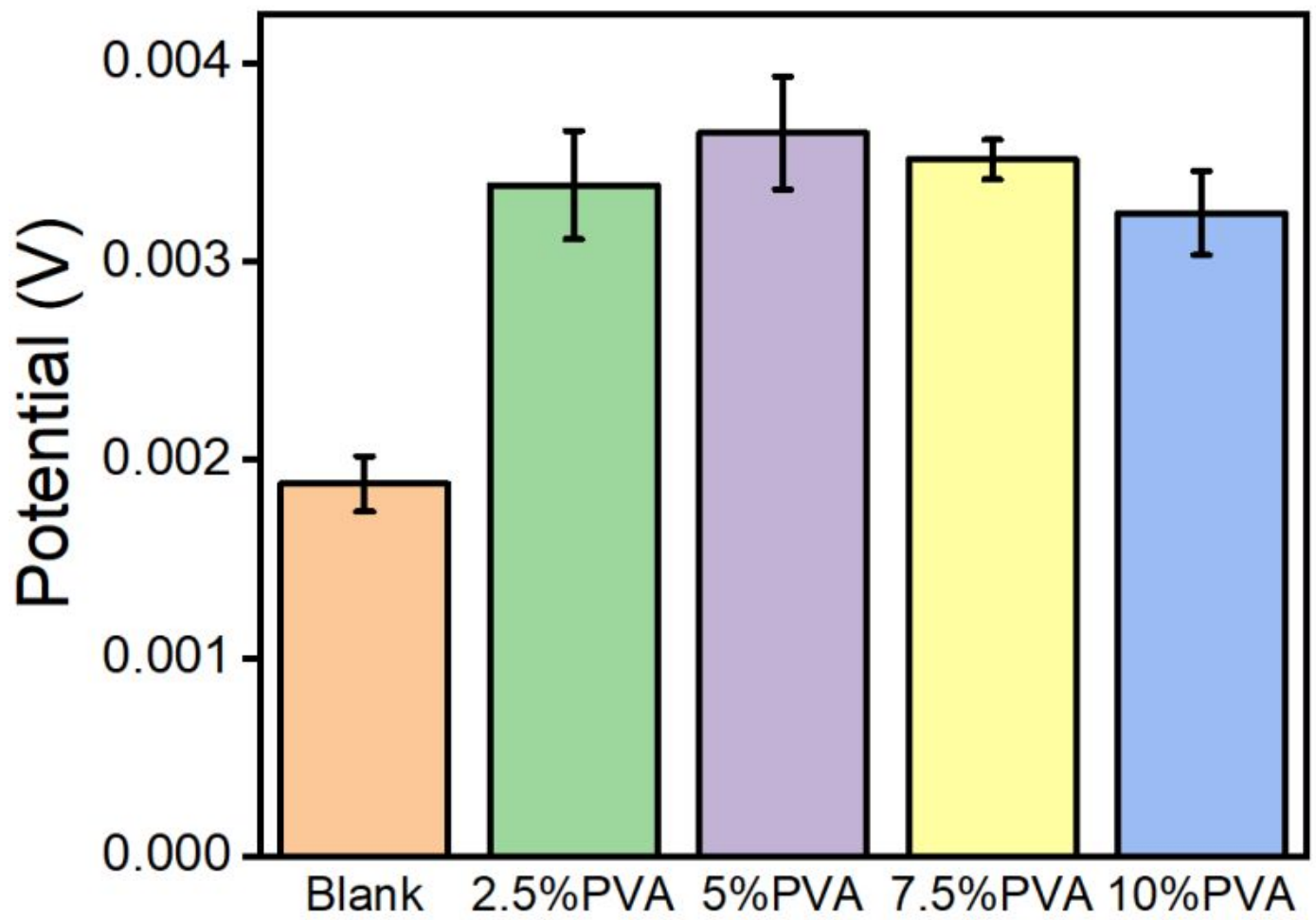

Figure S18. Potential difference generated by Peltier module with one side attached to PVA/NDI films prepared with varying PVA concentrations. Error bars calculated from standard deviation.

2.16 TGA curve of NDI and PVA/NDI films

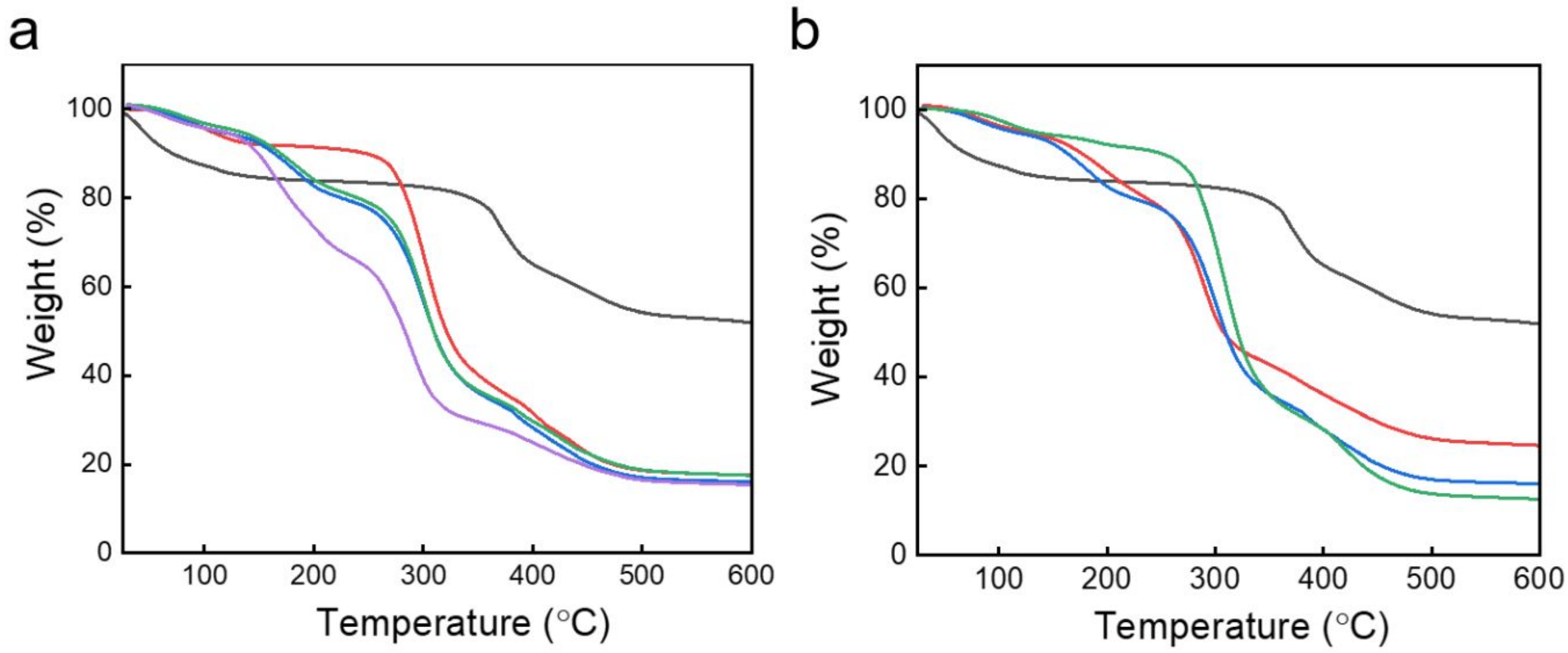

Figure S19. Thermogravimetric analysis (TGA) curves of PVA/NDI films from room temperature to 600 °C, showing the effects of glycerol (a) and PVA (b) concentrations. In (a), black: pure NDI-I; red: 5% PVA without glycerol; blue: 5% PVA + 0.5% glycerol; green: 5% PVA + 1% glycerol; purple: 5% PVA + 2% glycerol. In (b), black: pure NDI-I; red: 2.5% PVA + 0.5% glycerol; blue: 5% PVA + 0.5% glycerol; green: 10% PVA + 0.5% glycerol.

2.17 Mechanical Durability and Retention of Photothermal Behaviour of PVA/NDI-I Composite Films

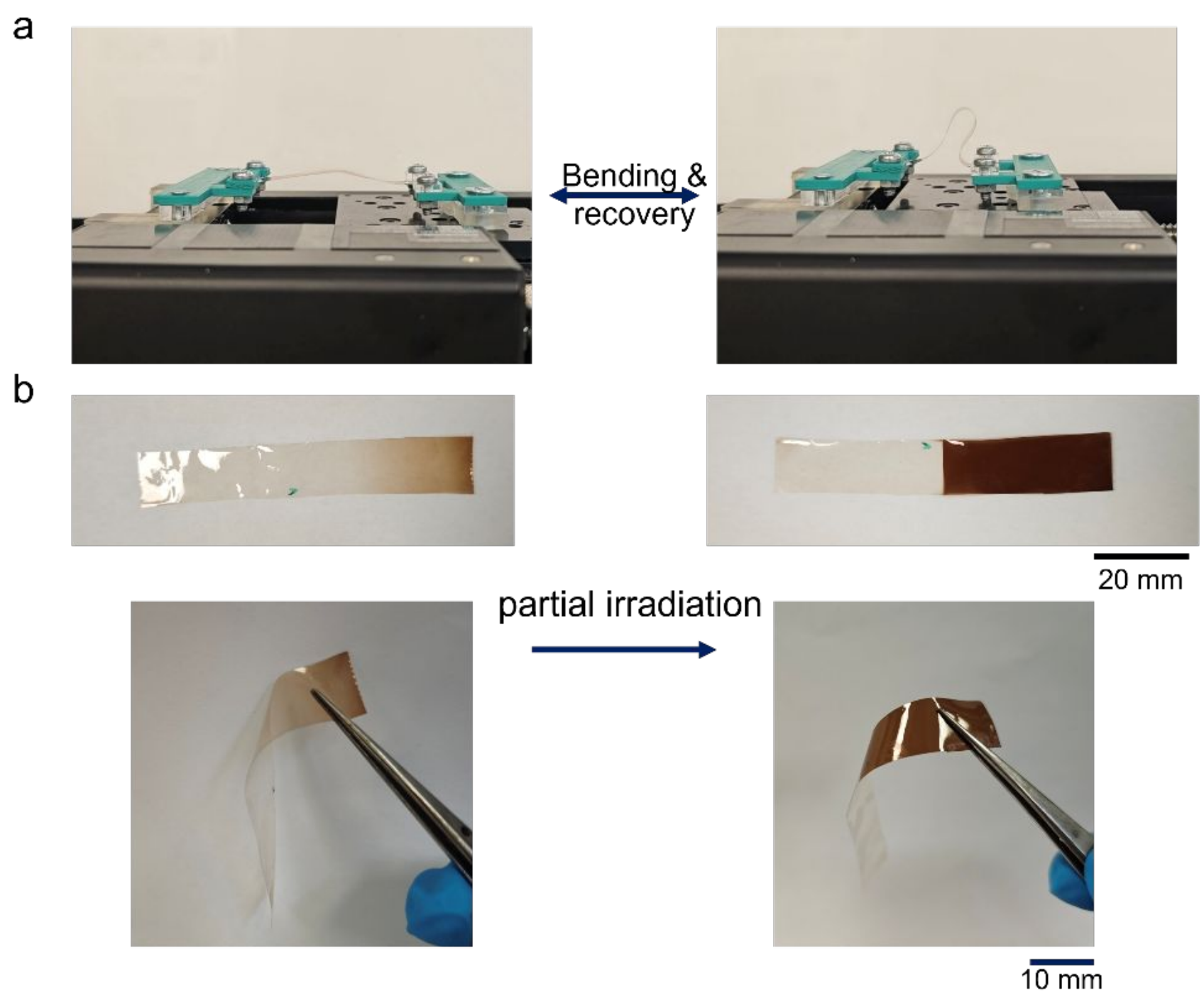

Figure S20. Mechanical flexibility and retention of photothermal behaviour of PVA/NDI composite films after repeated mechanical deformation. (a) Photographs of the composite film during the relaxation and bending stages of a single bending–relaxation cycle. (b) Photographs of the film after 1000 bending–relaxation cycles before and after partial irradiation.

3 References

(1) Xu, Z.; Doutch, J.; Mahmoudi, N.; Sproules, S.; Draper, E. R. On the photothermal behaviour of naphthalene diimide aggregates in water. *Journal of Materials Chemistry C* **2025**, *14*, 205–211. DOI: 10.1039/d5tc03120f.
